# Amphipolar, Amphiphilic 2,4-diarylpyrano[2,3-*b*]indoles as Turn-ON Luminophores in Acidic and Basic Media

**DOI:** 10.3390/molecules27072354

**Published:** 2022-04-06

**Authors:** Tobias Wilcke, Alexandru Postole, Marcel Krüsmann, Matthias Karg, Thomas J. J. Müller

**Affiliations:** 1 Institut für Organische Chemie und Makromolekulare Chemie, Heinrich-Heine-Universität Düsseldorf, Universitätsstrasse 1, D-40225 Düsseldorf, Germany; t.wilcke@hhu.de (T.W.); alexandru.postole@su.se (A.P.); 2 Institut für Physikalische Chemie, Heinrich-Heine-Universität Düsseldorf, Universitätsstrasse 1, D-40225 Düsseldorf, Germany; marcel.kruesmann@hhu.de (M.K.); karg@hhu.de (M.K.)

**Keywords:** dynamic light scattering, halochromicity, heterocycles, luminescence, sensors, UV/Vis spectroscopy

## Abstract

A versatile amphiphilic pyrano[2,3-*b*]indole for halochromic turn-ON luminescence in acidic or basic media is accessed by an insertion-coupling-cycloisomerization and adjusting solubilizing and phenolic functionalities. While almost non-emissive in neutral solutions, treatment with acids or bases like trifluoroacetic acid (TFA) or 1,8-diazabicyclo[5.4.0]undec-7-ene (DBU) reveals distinct luminescence at wavelengths of 540 nm or 630 nm in propan-2-ol, respectively. Turn-ON emission can be detected at pH values as mild as pH = 5.31 or 8.70. Quantum yields in propan-2-ol are substantial for protonated (*Φ_f_* = 0.058) and deprotonated (*Φ_f_* = 0.059) species. Photometrically, p*K_a_*_1_ of 3.5 and p*K_a_*_2_ of 10.5 were determined in propan-2-ol. With lipophilic polyether sidechains and hydrophilic protonation and deprotonation sites the molecule can be regarded as amphipolar, which results in good solubility properties for different organic solvents. In aqueous media, an organic co-solvent like propan-2-ol (35%) or tetrahydrofuran (25%) is needed, and the solution can be diluted with pure water without precipitation of the compound. At higher concentrations of water, a turbid solution is formed, which indicates the formation of micellar structures or clusters. With dynamic light scattering we could show that these clusters increase in size with increasing water content.

## 1. Introduction

Functional organic materials [1] have become increasingly important in many different fields of application, such as electronics [1,2,3,4,5,6,7,8,9,10,11,12,13], biomedicine [14,15,16,17,18,19], and analytics [20,21,22,23,24,25,26,27,28]. A rational design allows for the tailoring of the molecular property and applicability of these molecular materials [29,30,31,32,33,34,35,36,37,38]. In particular, structure-property relationships help with the understanding of the materials’ characteristics, thus improving their performance. Especially in the field of analytics, stimuli responsive molecules are of great interest due to their ability to selectively and reversibly bind an analyte, while exhibiting significant and detectable changes of their molecular electronic properties, e.g., color, luminescence, or redox potentials [39,40,41,42,43,44,45,46,47,48,49]. Analytes that can be detected by chemosensors are manifold, ranging from biologically relevant anions and cations to protons, gases and even small neutral molecules [50,51,52,53]. Functional organic materials with sensing properties include polymers, nanoparticles, as well as small heterocyclic molecules [28,54,55,56,57,58,59]. Heterocycles with stimuli responsive changes of their photophysical properties possess significant advantages over other read-out signals. Turn-ON luminescence, for instance, can be measured against a dark background and therefore offers a high sensitivity towards analytes. Moreover, fast read-outs, low costs, and comparably simple instrumentation are further advantages of these types of turn-ON luminescence chemosensors. Other possible photophysical read-outs include chromicity responses, i.e., changes in absorption or emission bands, as well as varying signal intensities or lifetime domains [60].

**Scheme 1:**
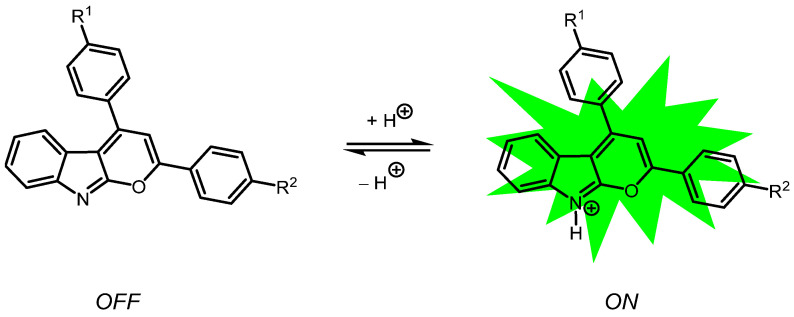
Reversibly switchable halochromic turn-ON luminescent 2,4-diarylpyrano[2,3-*b*]indoles.

Recently, we have demonstrated that 2,4-diarylpyrano[2,3-*b*]indoles are readily accessible from alkynoyl *ortho*-iodo anilides and terminal arylalkynes through a Pd-Cu-catalyzed domino insertion-coupling-cycloisomerization reaction [61,62]. Investigations of their photophysical properties revealed that halochromic luminescence in solution is induced through protonation in acid media or complexation with quantum yields of up to 15% (Scheme 1). Based on these observations, the use of 2,4-diarylpyrano[2,3-*b*]indoles as turn-ON luminophores and proton-sensitive chemosensors was considered, even expanding the solvent systems and the solubility [62].

Herein, we disclose a novel type of versatile amphiprotic 2,4-diarylpyrano[2,3-*b*]indole system suitable for the detection of both acids as well as bases through turn-ON luminescence in organic and aqueous solutions. Besides the synthesis, photophysical characterization and particle size analysis of an amphiprotic 2,4-diarylpyrano[2,3-*b*]indole are presented and discussed.

## 2. Results and Discussion

### 2.1. Synthesis of the 2,4-diarylpyrano[2,3-b]indole Chromophores

**Scheme 2:**
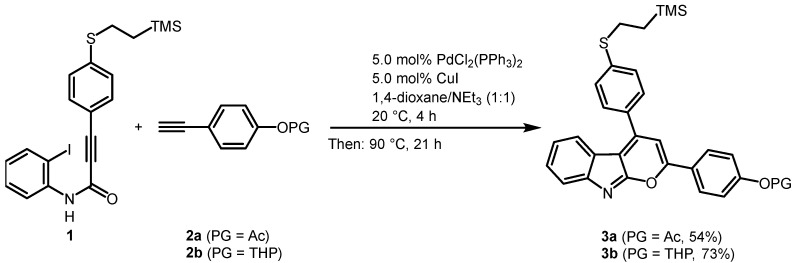
Pd-Cu-catalyzed domino synthesis of *O*-protected 2,4-diarylpyrano[2,3-*b*]indoles **3**.

Based on our previous synthetic protocols [61,62], a novel generation of halochromic 2,4-diarylpyrano[2,3-*b*]indoles containing a hydroxyl group has been created using our potassium fluoride mediated deprotection-alkylation one-pot reaction. Starting from secondary amide **1 [62]** and terminal alkyne **2a [63]**, the *O*-acyl protected 2,4-diarylpyrano[2,3-*b*]indole **3a** is formed by a Pd- and Cu-catalyzed domino insertion-coupling-cycloisomerization reaction in moderate yields of up to 54% (Scheme 2). Alternatively, 2,4-diarylpyrano[2,3-*b*]indole **3b** containing a 2-tetrahydropyranyl (THP) group can be synthesized by the coupling of alkyne **2b** in 73% yield.

**Scheme 3:**
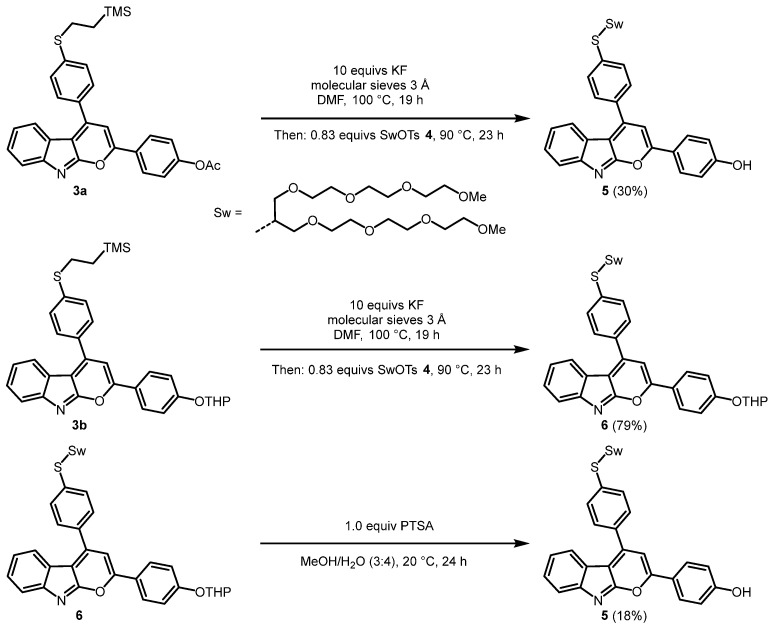
Deprotection-alkylation synthesis of 2,4-diarylpyrano[2,3-*b*]indole **5** starting from substrates **3a** and **3b**.

The solubilizing swallowtail substituent can be introduced by fluoride-induced desilylative cleavage of the (trimethylsilyl)ethyl group of 2,4-diarylpyrano[2,3-*b*]indoles **3** [62]. Interestingly, KF simultaneously acts as a desilylation and deacylation agent and for substrate **3a** (PG = Ac) both *O*- and *S*-protecting groups are concomitantly cleaved in the presence of an excess of potassium fluoride and the subsequent addition of the swallowtail tosylate **4** selectively leads *S*-alkylation furnishing hydroxy 2,4-diarylpyrano[2,3-*b*]indole **5** in 30% yield in a one-pot fashion (Scheme 3). Activated molecular sieves, added at the beginning of the reaction, prove to be beneficial to maintain the thiolate intermediate, which is sensitive against moisture. In contrast, substrate **3b** (PG = THP) is transformed to the targeted hydroxy 2,4-diarylpyrano[2,3-*b*]indole **5** in two separate steps: first the desilylative *S*-alkylation with the swallowtail tosylate **4** in 79% yield and then a PTSA-mediated deprotective transacetalization [64] of the THP-protected *S*-alkyl derivative **6** furnishes the target compound **5** in 18% yield. Due to the lower overall yield of the last step, the first synthetic route starting from acyl-protected 2,4-diarylpyrano[2,3-*b*]indole **3a** is preferred. Also, this route is more elegant, since deprotection of the hydroxyl group takes place in the same reaction vessel. For further investigations, no distinctions were made between the products of the two different synthetic routes.

**Scheme 4:**
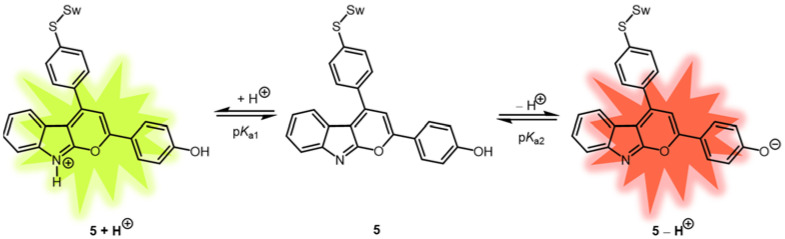
Protonation (left) and deprotonation (right) of amphoteric 2,4-diarylpyrano[2,3-*b*]indole **5**.

**Figure 1:**
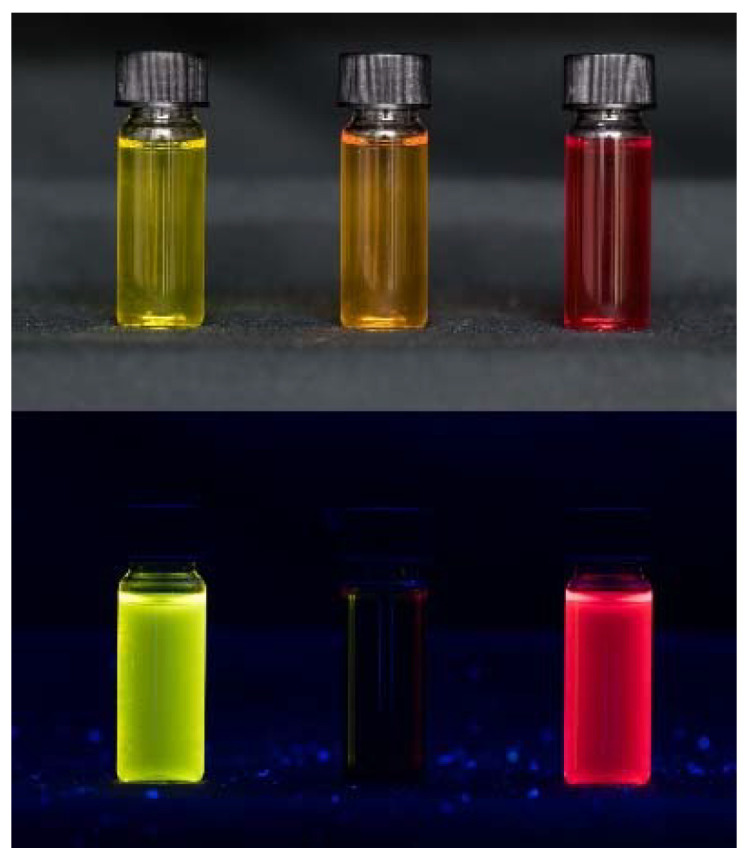
Halochromic solutions (*c* = 1.3 × 10^−3^ mol·L^−1^) of 2,4-diarylpyrano[2,3-*b*]indole **5** in propan-2-ol under daylight (top row) and UV-light (*λ*_exc_ = 354 nm) (bottom row). The solution protonated **5 + H^+^** with TFA (left), the neutral solution of **5** (center), and the solution deprotonated **5 − H^+^** with DBU (right).

Similarly as 2,4-diarylpyrano[2,3-*b*]indoles of the first [61] and second [62] generation, 2,4-diarylpyrano[2,3-*b*]indole **5** is also non-luminescent in the solid state and solution. Upon protonation with stronger acids, such as trifluoroacetic acid (TFA), solutions of 2,4-diarylpyrano[2,3-*b*]indole **5** exhibit yellow luminescence upon excitation with UV-light (Scheme 4, Figure 1 left). A similar effect was previously reported after adding metal cations, such as Zn^2+^ or Mg^2+^, which are complexed by 2,4-diarylpyrano[2,3-*b*]indole **5** [61].

In addition, 2,4-diarylpyrano[2,3-*b*]indole **5** shows a peculiar response to deprotonation of the phenolic hydroxy group in solution with stronger bases, such as 1,8-diazabicyclo[5.4.0]undec-7-ene (DBU), which to this date has not been reported for this type of class. Upon deprotonation in solution, the resulting phenolate oxygen exerts a very strongly electron donating character, leading to an extended *π*-conjugated push-pull system displaying a bright red emission (Scheme 4, Figure 1 right). It is worthy of mention, that both protonation and deprotonation are reversible upon the addition of TFA or DBU, respectively.

### 2.2. Solubility of 2,4-diarylpyrano[2,3-b]indole ***5***

Next, the solubility of 2,4-diarylpyrano[2,3-*b*]indole **5** was scrutinized. Hereby, the focus was laid on identifying suitable solvents and solvent systems, where 2,4-diarylpyrano[2,3-*b*]indole **5** and its photophysical properties as well as other effects could be properly investigated.

Starting at a given concentration (*c* = 1.3 × 10^−3^ mol·L^−1^), the mixture of 2,4-diarylpyrano[2,3-*b*]indole **5** and corresponding solvent were stirred for 30 min and sonicated for 10 min. Solubility upon eyesight was indicated with “+”, whereas the presence of undissolved compound was indicated with “–”. At first, organic solvents from lesser to higher polarity according to Reichardt’s polarity scale [65] were employed (Table 1). Specifically, non-toxic solvents were of interest, opening the possibility for an application as a halochromic chemosensor for protons and the absence of such in biological systems.

As seen in Table 1, (un)branched alkanes like *n*-pentane and unbranched ethers like 2-methoxy-2-methylpropane (MTBE) are not suitable for solubilizing 2,4-diarylpyrano[2,3-*b*]indole **5**, whereas other non-polar solvents like benzene and toluene are able to dissolve 2,4-diarylpyrano[2,3-*b*]indole **5** completely. This can be plausibly rationalized by *π*-*π* interactions between molecules **5** and the aromatic solvent, preventing them from aggregation. More polar solvents, such as 1,4-dioxane, acetone, and propan-2-ol, are all suitable for further investigations, as well as chlorinated solvents, such as chloroform.

Most of the solvents able to dissolve chromophore **5** lead to intense luminescence after addition of an excess of TFA observed by the naked eye, but only a few solvents enable luminescence in the deprotonated state. Mainly protic solvents of higher polarity, such as propan-2-ol, ethanol or methanol, show noticeable luminescence, thus narrowing the applicable solvent spectrum for switching in the polarity range. For further photophysical investigations, propan-2-ol is employed as a protic solvent with the most intense luminescence in both protonated and deprotonated states for the naked eye.

Unfortunately, water alone, as the most polar protic solvent, is not able to solubilize chromophore **5**. Therefore, water mixtures with different polar co-solvents such as THF and propan-2-ol were prepared as well as selected aqueous systems and mixtures of compound **5** at different pH values (Table 2). Again, solubility at a specified concentration (*c* = 1.3 × 10^−3^ mol·L^−1^) was indicated with “+”, whereas the presence of undissolved compound was indicated with “–”.

While biologically important buffers [66,67] like HEPES (2-[4-(2-hydroxyethyl)piperazin-1-yl]ethanesulfonic acid) or MOPS (3-morpholinopropane-1-sulfonic acid) did not facilitate the water solubility of dye **5**, a clear trend can be identified for aqueous systems at different pH values (Table 2). The more basic or acidic the solution, the better dye **5** is soluble. Below pH 3 and above pH 11, complete solubility was achieved. This can be rationalized by the structure and functional groups of 2,4-diarylpyrano[2,3-*b*]indole **5**. Below pH 3, complete protonation at the nitrogen atom of the indole moiety occurs and the positive charge causes repulsion and, hence, less *π*-*π*-stacking between the molecules and better solubility in aqueous media. The same principle holds true for aqueous solutions with pH >11. Through deprotonation with a strong organic base such as DBU, a phenolate is created, resulting in complete dissolving of dye **5** under these conditions. However, at higher pH values above 13, degradation of the 2,4-diarylpyrano[2,3-*b*]indoles can be observed [62]. Moreover, the p*K*_a1_ value for indole nitrogen atom protonation and the p*K*_a2_ value for phenol deprotonation can be estimated by the solubility. Since solubility starts below pH 3 and above pH 11, a p*K*_a1_ value of around 3 and p*K*_a2_ of around 11 can be roughly assumed. This qualitative estimation can be quantified by UV/Vis photometric determination of both p*K*_a_ values (see Section 2.4).

**Figure 2:**
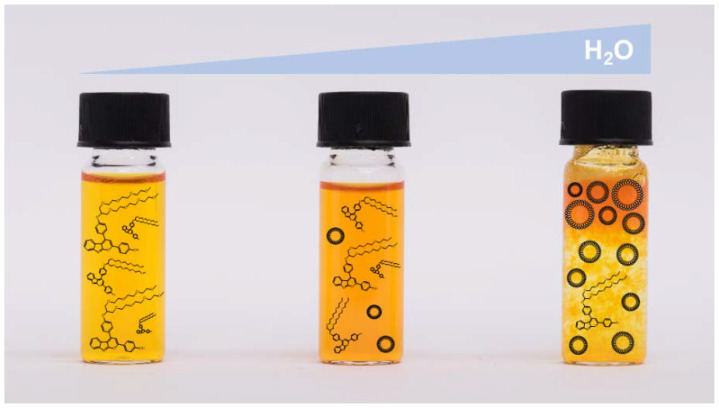
2,4-Diarylpyrano[2,3-*b*]indole **5** in propan-2-ol with increasing water fractions from left (0%) and middle (70%) to right (95%). In the vial to the right cluster formation is clearly visible.

For the mixtures of organic co-solvent and water, 2,4-diarylpyrano[2,3-*b*]indole **5** is dissolved in each organic solvent and this solution is rapidly injected in the corresponding amount of water. In Table 2, the lowest amount of co-solvent, which is needed for solubilizing 2,4-diarylpyrano[2,3-*b*]indole **5** in aqueous media is presented. Hereby, the fraction of water is incrementally increased in steps of 5 vol-% until formation of clusters or turbid solutions can be observed indicating too high and non-suitable water fractions (Figure 2). At excessive fractions, the formation of clusters occurs rapidly just seconds after the mixing of the two solvents. Since we expect that size and appearance of the clusters depend strongly on the solvent mixture, we used dynamic light scattering (DLS) to analyze the different samples.

### 2.3. Particle size of 2,4-diarylpyrano[2,3-b]indole ***5*** in Aqueous Solutions

**Figure 3:**
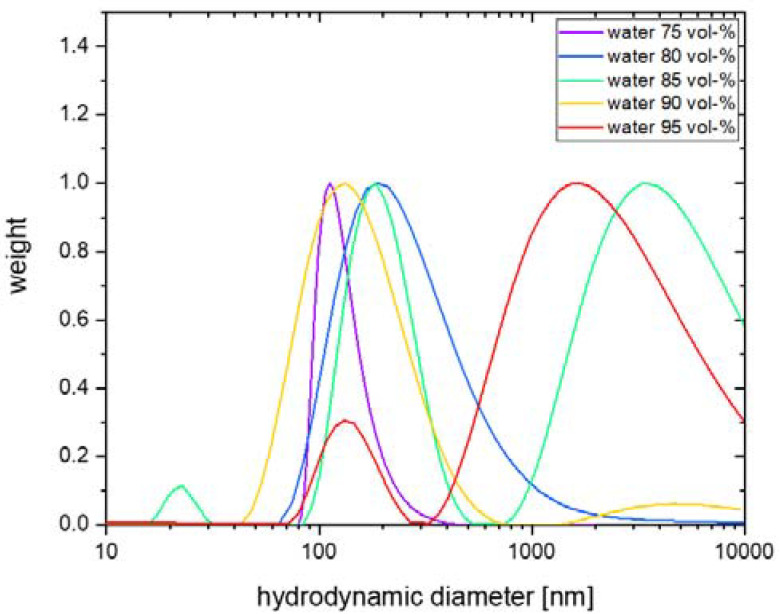
Size distribution of the 2,4-diarylpyrano[2,3-*b*]indole **5** cluster in different propan-2-ol water mixtures obtained from CONTIN analysis of DLS data.

DLS was used to study the hydrodynamic size and size distribution with regard to the dependence of the water content in the solvent mixture. For this 2,4-diarylpyrano[2,3-*b*]indole **5** was dissolved in different propan-2-ol/water mixtures. The water content of the mixtures was varied from 75 up to 95 vol-% in 5 vol-% steps. All samples were sonicated for 30 min and filtered using a filter with 5 µm pore size before the DLS measurement. This was mostly done to remove dust and potentially very large impurities or aggregates. We want to highlight that the filtered samples did not show any precipitation in the course of several hours, i.e., much longer than the measurement times in DLS. The size distributions of all measured samples were obtained at a 110° scattering angle by CONTIN analysis [68] are shown in Figure 3.

At the lowest water concentration of 75 vol-% (purple) we find a monomodal size distribution with an average cluster size of approximately 140 nm in diameter. The narrow width of the distribution indicates rather low polydispersity. In contrast, when increasing the water content, the size distribution of the clusters shifts to larger sizes with increased polydispersity. For water contents of 85 vol-% and above, additional contributions become visible and the distribution functions become at least bimodal with a contribution from larger aggregates in the micrometer regime. Nevertheless, even these samples were found to be colloidally stable, at least in the course of hours. The size of the aggregates varies strongly between samples and cannot be precisely determined. In particular, the sample at a water concentration of 95 vol-% contains large and polydisperse aggregates that dominate the light scattering. This finding is in good agreement with our turbidity observations previously shown in this work. Nevertheless, we attempted analysis from angle-dependent DLS (see Figure S19, Supporting Information) to study the diffusive behavior of the smaller cluster phase. For these we find translational diffusion that allowed for a more quantitative size analysis. The results are reported in Table S3 (Supporting Information).

In addition, the stability of the clusters and aggregates over time was investigated. For this the measurements were repeated after one month. We found a significant increase in size and polydispersity after this aging time (see Figure S20, Supporting Information). This is a strong indication that the clusters are not stable in these solvent mixtures over a long period of time.

### 2.4. Photophysical Properties of 2,4-diarylpyrano[2,3-b]indole ***5***

**Figure 4:**
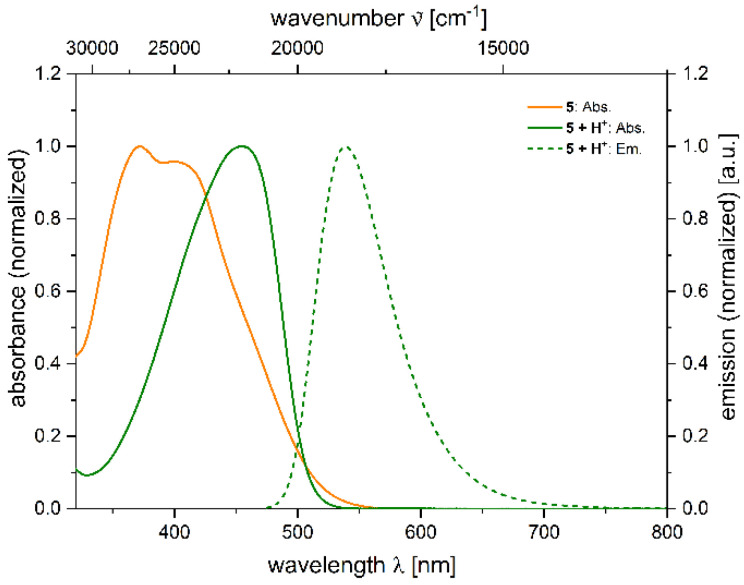
Normalized absorption spectrum of **5** (orange solid line), **5 + H^+^** (green solid line), and normalized emission spectrum of **5 + H^+^** (green dashed line) (*c*_0_(**5**) = 5.0 × 10^−5^ mol·L^−1^, *c*(TFA) = 10^−2^ mol·L^−1^, recorded in propan-2-ol at *T* = 293 K, *λ*_exc,**5 + H**_**^+^** = 455 nm).

**Figure 5:**
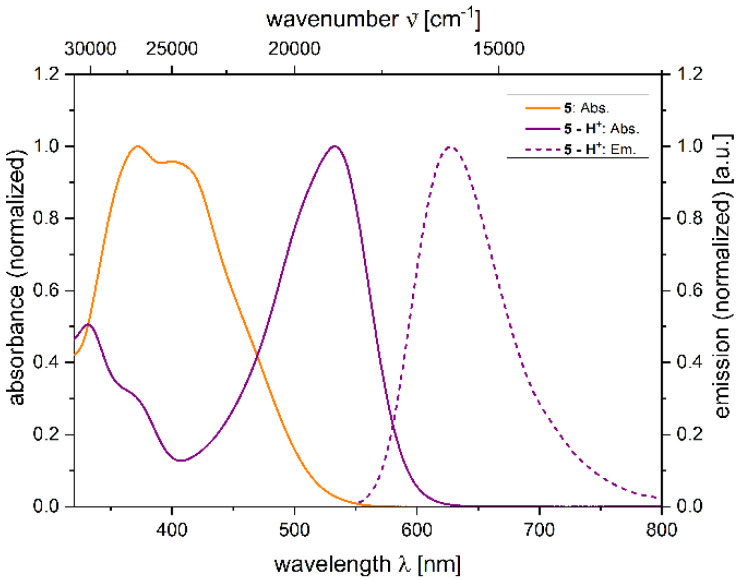
Normalized absorption spectrum of **5** (orange solid line), **5 − H^+^** (purple solid line), and normalized emission spectrum of **5 − H^+^** (purple dashed line) (*c*_0_(**5**) = 5.0 × 10^−5^ mol·L^−1^, *c*(DBU) = 10^−2^ mol·L^−1^, recorded in propan-2-ol at *T* = 293 K, *λ*_exc,**5 − H**_**^+^** = 533 nm).

Although non-luminescent in the solid state and in solution, 2,4-diarylpyrano[2,3-*b*]indole **5** displays peculiar luminescence under certain conditions, rendering it a turn-ON luminophore sensitive to acidic and basic solutions as well as for metal cations. Herein, the photophysical properties of 2,4-diarylpyrano[2,3-*b*]indole **5** in propan-2-ol under neutral, acidic, and basic conditions are discussed (Table 3, Figure 4 and Figure 5).

In the ground state, the neutral dye **5** shows an expected absorption behavior comparable to 2,4-diarylpyrano[2,3-*b*]indoles with an 4-anisyl group at position 2 (*λ_max,abs_* = 369 nm) [62]. Upon protonation, the absorption maximum shifts from 372 to 455 nm. Moreover, the absorption maximum can be shifted even further bathochromically upon deprotonation, i.e., the absorption maximum is shifted from 372 to 533 nm. Instead of a cyanine chromophore in the acidic medium, an oxonol anion chromophore is formed in the basic medium. The molar decadic absorption coefficients *ε* of 2,4-diarylpyrano[2,3-*b*]indole **5** increase upon (de)protonation from 14,200 L·cm^−1^·mol^−1^ for the neutral species **5** to 24,500 L·cm^−1^·mol^−1^ for the deprotonated species **5 − H^+^** and 22,400 L·cm^−1^·mol^−1^ for the protonated species **5 + H^+^**. Likewise, the emission maxima shift to longer wavelengths for the ionized forms of 2,4-diarylpyrano[2,3-*b*]indole **5** with similar Stokes shifts of 3400 cm^−1^ (**5 + H^+^**) and 2800 cm^−1^ (**5 − H^+^**). The absolute quantum yields *Φ_f_* for both species were measured with an Ulbricht sphere and reach up to *Φ_f_* = 0.058 (**5 + H^+^**) and *Φ_f_* = 0.059 (**5 − H^+^**).

As seen in Table 2, 2,4-diarylpyrano[2,3-*b*]indole **5** is soluble in mixtures of water and propan-2-ol up to 65:35 vol-%, raising the question, how the photophysical properties are affected by water as a solvent. After replacing about 2/3 of the organic solvent by water, molar decadic absorption coefficients *ε* for all 2,4-diarylpyrano[2,3-*b*]indole species **5**, **5 + H^+^**, and **5 − H^+^** increase up to 70% (Table 4).

**Figure 6:**
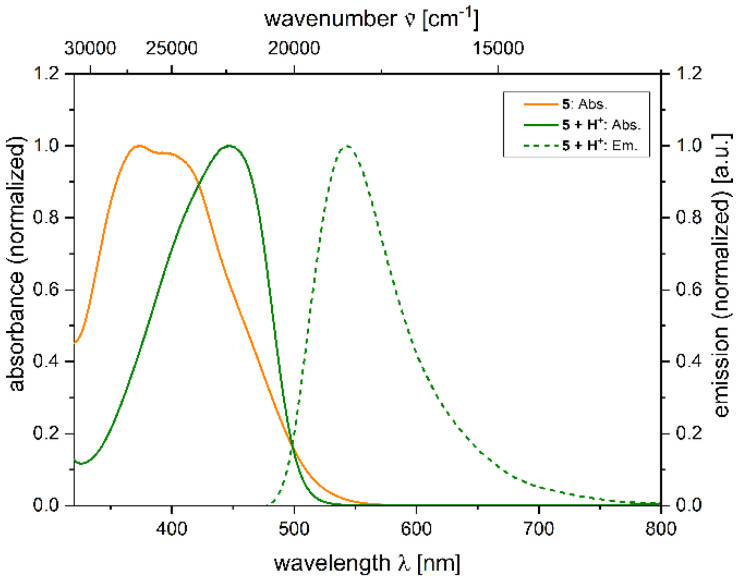
Normalized absorption spectra of **5** (orange solid line), **5 + H^+^** (green solid line), and normalized emission spectrum of **5 + H^+^** (green dashed line) (*c*_0_(**5**) = 5.1 × 10^−5^ mol·L^−1^, recorded in propan-2-ol/H_2_O (35:65) at *T* = 293 K; *λ*_exc,**5 + H**_**^+^** = 447 nm).

**Figure 7:**
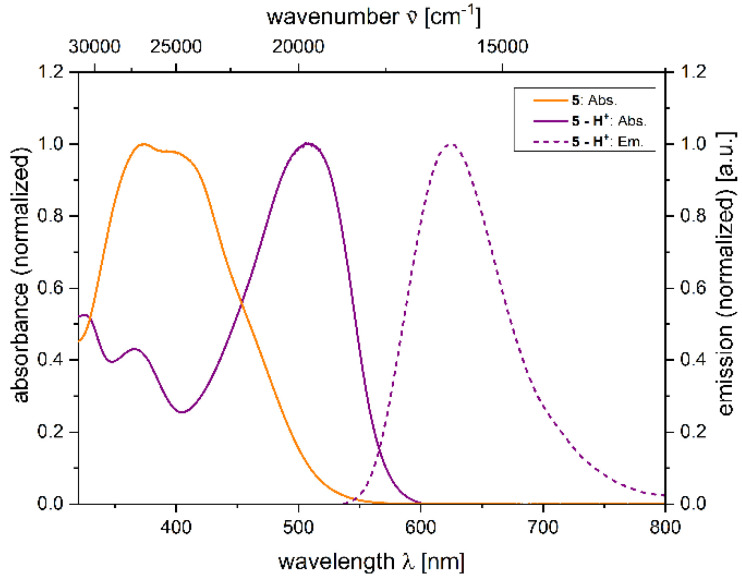
Normalized absorption spectra of **5** (orange solid line), **5 − H^+^** (purple solid line), and normalized emission spectrum of **5 − H^+^** (purple dashed line) (*c*_0_(**5**) = 5.1 × 10^−5^ mol·L^−1^, recorded in propan-2-ol/H_2_O (35:65) at *T* = 293 K; *λ*_exc,**5 − H**_**^+^** = 509 nm).

While emission bands of **5 + H^+^** and **5 − H^+^** remain at the same wavelength as in pure propan-2-ol (Figure 6 and Figure 7), absorption bands are slightly shifted hypsochromically, resulting in larger Stokes shifts up to Δ*ῦ* = 4000 cm^−1^. Expectedly, the absolute quantum yields of **5 + H^+^**, and **5 − H^+^** diminish and amount to *Φ_f_* = 0.014 and *Φ_f_* = 0.018, respectively, in propan-2-ol/water (35:65). Above a water fraction of 65%, formation of clusters can be observed leading to poorly resolved and noisy UV/Vis spectra.

For chemosensors, the analyte as well as the spectrum of action are essential and define their possible application. In the case of 2,4-diarylpyrano[2,3-*b*]indole **5**, besides metal cations, the main analytes are acidic and basic solutions due to its amphiprotic character. For amphiprotic molecules, the spectrum of action is defined by its two p*K*_a_ values, which can be determined by UV/Vis absorption titration and their change can be monitored in the presence of an analyte.

For the determination of p*K*_a1_ of 2,4-diarylpyrano[2,3-*b*]indole **5**, absorption spectra at various pH values in a range from 7 to 1.3 can be recorded upon addition of TFA in propan-2-ol. Propan-2-ol as a solvent is completely miscible with both TFA and DBU and the spectra are less noisy in comparison to other solvents, such as THF. In addition, propan-2-ol/water is also an aqueous system, where 2,4-diarylpyrano[2,3-*b*]indole **5** is soluble and, thus, can be employed as chemosensor.

**Figure 8:**
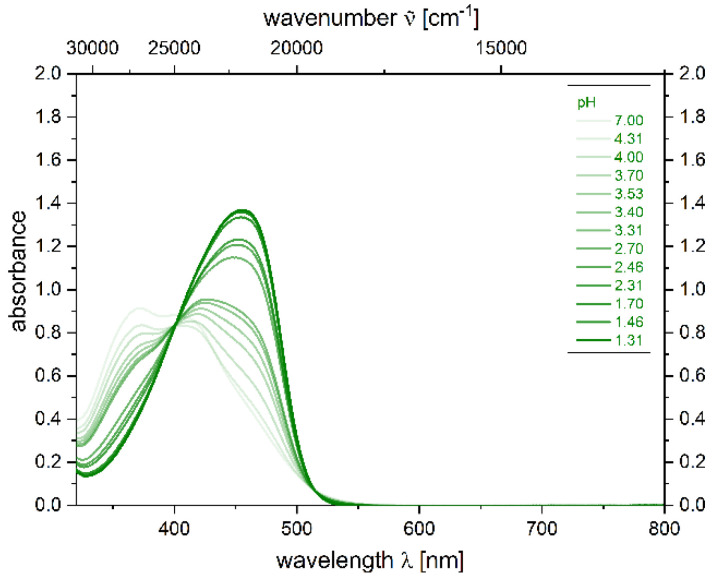
Absorption spectra of **5** (*c_0_*(**5**) = 5.0 × 10^−5^ mol·L^−1^) at different concentrations of trifluoroacetic acid (recorded in propan-2-ol at *T* = 293 K).

The spectra at different pH values display isosbestic points, indicating that deprotonation proceeds directly without an intermediate (Figure 8) [70]. The involved species are **5** and **5 + H^+^** furnishing a p*K*_a1_ value of 3.5 for the deprotonation of **5 + H^+^** (for details, see Supporting Information).

**Figure 9:**
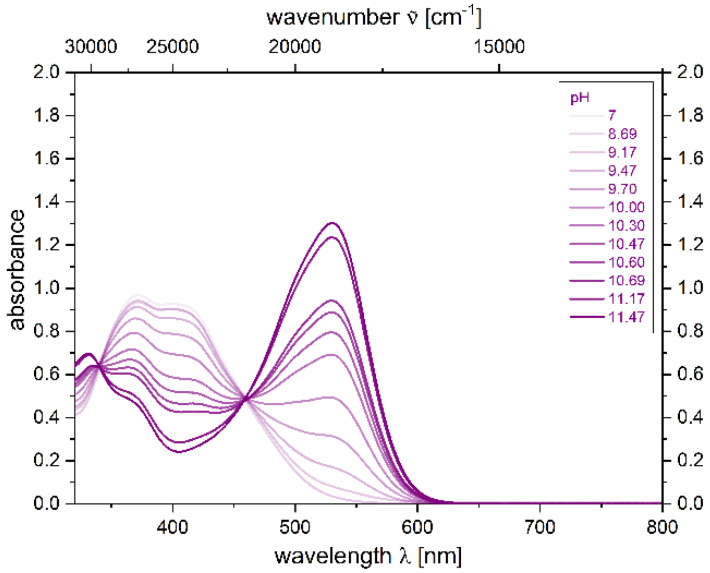
Absorption spectra of **5** (*c_0_*(**5**) = 5.0 × 10^−5^ mol·L^−1^) at different concentrations of 1,8-diazabicyclo[5.4.0]undec-7-ene (DBU) (recorded in propan-2-ol at *T* = 293 K).

Likewise, the p*K*_a2_ value of the phenolic hydroxy group can be determined by titration with DBU as a base. Again, the occurrence of isosbestic points indicates the direct transition from species **5** to **5 − H^+^** without an intermediate (Figure 9) furnishing the corresponding p*K*_a_ value of 10.5 for the deprotonation of **5** (for details, see Supporting Information). The average of both p*K*_a_ values accounts for an isoelectric point of pH = 7 for compound **5**. The calculated isoelectric point is in agreement with previous observations that neutral 2,4-diarylpyrano[2,3-*b*]indole **5** is insoluble in water at this pH region and facilitates aggregation.

**Figure 10:**
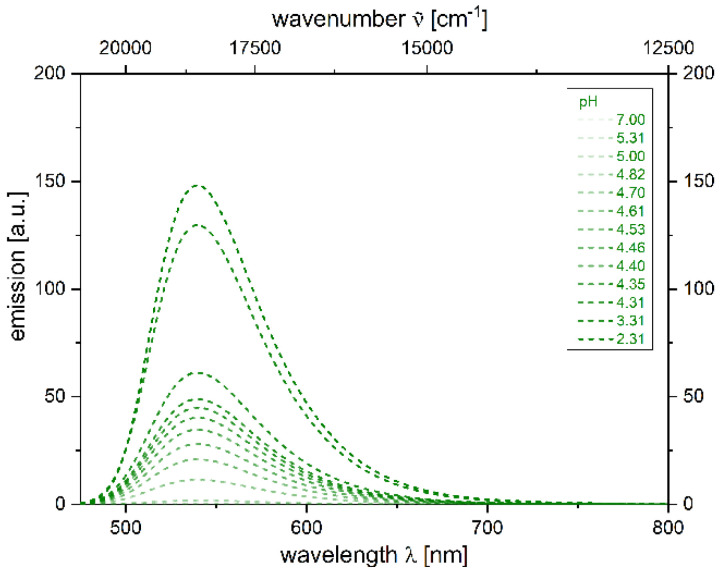
Emission spectra of **5** (*c_0_*(**5**) = 5.0 × 10^−5^ mol·L^−1^) at different concentrations of trifluoroacetic acid (recorded in propan-2-ol at *T* = 293 K).

**Figure 11:**
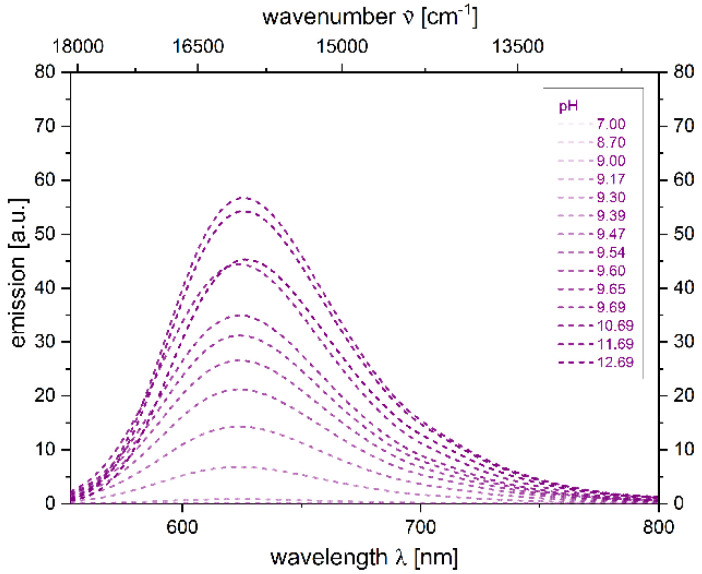
Emission spectra of **5** (*c_0_*(**5**) = 5.0 × 10^−5^ mol·L^−1^) at different concentrations of 1,8-diazabicyclo[5.4.0]undec-7-ene (DBU) (recorded in propan-2-ol at *T* = 293 K).

Furthermore, the emission properties at different pH values can be investigated. Upon titration, the pH detection limit under acidic and basic conditions is first determined (Figure 10 and Figure 11). For the addition of TFA, luminescence is turned on at pH values smaller than 5.31, as for the addition of DBU, luminescence turns on at pH values higher than 8.70. This leads to a blind spot of the chemosensor **5** between pH 5.31–8.70, where no significant emission takes place.

**Figure 12:**
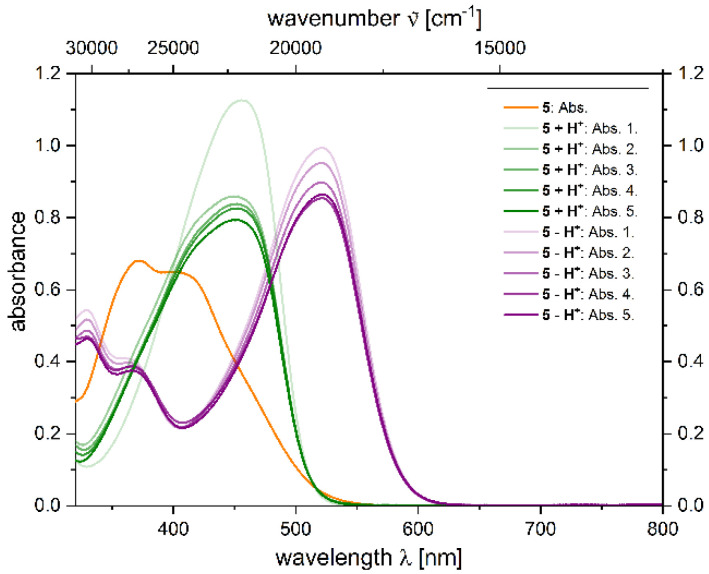
Absorption spectra of **5** (*c_0_*(**5**) = 3.5 × 10^−5^ mol·L^−1^, pH = 1.5–12.5) after alternating addition of trifluoroacetic acid and 1,8-diazabicyclo[5.4.0]undec-7-ene (DBU) (recorded in propan-2-ol at *T* = 293 K).

Finally, the proper reversibility after binding an analyte and, thus, the accuracy of the results have to be ascertained. For 2,4-diarylpyrano[2,3-*b*]indole **5**, the reversibility is tested by an alternating addition of an excess amount of TFA and DBU to a neutral solution of 2,4-diarylpyrano[2,3-*b*]indole **5** in propan-2-ol. After the first cycle of addition of TFA, DBU, and again TFA to 2,4-diarylpyrano[2,3-*b*]indole **5**, a decrease in absorbance can be seen (Figure 12), probably due to ion strength of [DBU-H^+^][TFA^−^] and its interaction with the chromophore. Furthermore, a comparably smaller decrease in absorbance after each cycle can be attributed to dilution by increasing the amount of acid and base, respectively. The color change during these cycles occurs considerably fast, allowing a rapid readout just in seconds after addition of the analyte. After five cycles, no further effects besides small decreases in absorbance can be seen and, therefore, a good reversibility of this chemosensor can be concluded.

## 3. Materials and Methods

### 3.1. General Considerations

Commercially available chemicals were purchased reagent grade and used without further purification. *N*-(2-iodophenyl)-3-(4-((2-(trimethylsilyl)ethyl)thio)phenyl)propiolamide (**1**) [62], 4-ethynylphenyl acetate (**2**) [63,71,72], swallowtail-tosylate (Sw-OTs, **4**) [71,72,73], and 2-(4-ethynylphenoxy)tetrahydro-2*H*-pyran (**6**) [74,75], were prepared according to literature protocols. Reactions were performed in oven-dried Schlenk tubes under a nitrogen atmosphere unless stated otherwise. Dry solvents were dried through a solvent purification system (MB-SPS-800, M. Braun Inertgas-Systeme, Garching, Germany). Reaction progress was qualitatively monitored using silica gel layered aluminium foil (60 F_254_, Merck, Kenilworth, NJ, USA). For detection, UV light of wavelengths 254 nm and 365 nm as well as an aqueous potassium permanganate solution was used. Column chromatography was performed using flash technique under pressure of 2 bar with silica gel 60, mesh 230-400 (Macherey-Nagel, Düren, Germany). ^1^H and ^13^C NMR spectra were measured on an Avance III-300 (Bruker, Billerica, MA, USA) and Avance III-600 (Bruker, Billerica, MA, USA) spectrometer. Chemical shifts (*δ*) were referenced to the internal solvent signal: CDCl_3_ (^1^H *δ* 7.26, ^13^C *δ* 77.2). Multiplicities are stated as: s (singlet), d (doublet), dd (doublet of doublet), t (triplet), and m (multiplet). Coupling constants (*J*) are given in Hz. The assignment of primary (CH_3_), secondary (CH_2_), tertiary (CH) and quaternary carbon nuclei (C_quat_) was made using DEPT-135 spectra. Mass spectrometric investigations were carried out at the Department of Mass Spectrometry of the Institute of Inorganic and Structural Chemistry, Heinrich-Heine-Universität Düsseldorf. IR spectra were recorded using an IR Affinity-1 spectrometer (Shimadzu, Kyoto, Japan) via the attenuated total reflection (ATR) method. The intensities of the IR bands are abbreviated as w (weak), m (medium), s (strong). Melting points (uncorrected) were measured using a Melting Point B-540 (Büchi, Oldham, UK). Combustion analyses were measured on a Series II Analyser 2400 (Perkin Elmer, Waltham, MA, USA) at the Institute of Pharmaceutical and Medicinal Chemistry, Heinrich-Heine-Universität Düsseldorf. Absorption spectra were recorded on a UV/Vis/NIR Lambda 19 spectrometer (Perkin Elmer, Waltham, MA, USA). Molar extinction coefficients *ε* were determined by absorption measurements at five different concentrations. Emission spectra were recorded using a F-7000 spectrometer (Hitachi, Tokyo, Japan) and corrected with spectra of the solvent mixtures. Excitation slit was set to 5.0 nm and emission slit was set to 10 nm. PMT Voltage was set to 350 V and scan speed was set to 240 nm/min. Absolute quantum yields were determined using a Spectrofluorometer FS5 (Edinburgh Instruments, Livingston, UK). Light scattering experiments were performed on a 3D LS Spectrometer (LS Instruments, Fribourg, Switzerland) in 2D mode. A HeNe-Laser was used as a light source and two Avalanche photodiodes in cross-correlation mode were used for the detection of scattered light. The samples were placed in a refractive index matching bath filled with decalin. The temperature control was realized with a JULABO CF31 and a PT100 probe near the sample position. The samples were prepared in Fisherbrand culture tubes (Fisher Scientific GmbH, Schwerte, Germany) with an outer diameter of 1 cm. Measurements were performed over a range of scattering angles of 30° to 140° in 10° steps. Three measurements with acquisition times between 10 and 60 s, depending on the scattering intensity of the sample, were performed at each angle. The temperature for all measurements was set to 25 °C. The data were analyzed using the CONTIN algorithm [68] as implemented in AfterALV v.1.0e (Dullware), The Netherlands.

### 3.2. Synthesis of 4-(4-(4-((2-(trimethylsilyl)ethyl)thio)phenyl)pyrano[2,3-b]indol-2-yl)phenyl acetate *(**3a**)*

To a degassed solution of **1** (1.38 g, 2.88 mmol) in triethylamine/1,4-dioxane (1:1, 28 mL) was added PdCl_2_(PPh_3_)_2_ (0.101 g, 0.144 mmol), CuI (0.027 g, 0.14 mmol), and alkyne **2a** (0.508 g, 3.17 mmol) and the mixture was stirred at 20 °C for 4 h. Then, the reaction mixture was stirred at 90 °C for another 18 h. After cooling to room temp, the mixture was diluted with water (50 mL) and extracted with ethyl acetate (3 × 100 mL). The combined organic layers were then washed with a saturated solution of aqueous NH_4_Cl (2 × 100 mL) and dried with anhydrous Na_2_SO_4_. After filtration, the solvent was removed under reduced pressure and the crude product was purified by column chromatography on silica gel (cyclohexane/ethyl acetate (1:1)) and recrystallized in *n*-hexane/ethyl acetate (3:1) to give compound **3a** (0.815 g, 1.59 mmol, 54%) as a dark red solid, Mp 90 °C. R*_f_* (*n*-hexane/acetone 1:1) = 0.30.

^1^H NMR (300 MHz, CDCl_3_): *δ* 0.11 (s, 9 H), 1.01–1.07 (m, 2 H), 2.35 (s, 3 H), 3.08–3.14 (m, 2 H), 7.11–7.16 (m, 2 H), 7.29 (d, *^3^J_H-H_* = 8.7 Hz, 2 H), 7.47–7.52 (m, 3 H), 7.73–7.81 (m, 4 H), 8.10 (d, *^3^J_H-H_* = 8.7 Hz, 2 H). ^13^C NMR (75 MHz, CDCl_3_): *δ* −1.6 (CH_3_), 16.7 (CH_2_), 21.3 (CH_3_), 28.7 (CH_2_), 104.8 (CH), 118.7 (C_quat_), 119.3 (CH), 121.8 (CH), 122.4 (CH), 122.5 (C_quat_ ), 122.6 (CH), 127.5 (CH), 127.8 (CH), 129.0 (CH), 129.3 (CH), 129.3 (C_quat_), 132.9 (C_quat_), 141.9 (C_quat_), 146.6 (C_quat_), 151.6 (C_quat_), 152.9 (C_quat_), 155.4 (C_quat_), 165.1 (C_quat_), 169.1 (C_quat_). IR (ATR):*ῦ* [cm^−1^] 2951 (w), 1755 (m), 1636 (m), 1593 (w), 1541 (m), 1522 (s), 1503 (s), 1472 (m), 1437 (m), 1414 (m), 1368 (m), 1321 (w), 1302 (w), 1281 (w), 1256 (w), 1246 (m), 1188 (s), 1165 (s), 1125 (m), 1109 (m), 1098 (m), 1086 (m), 1070 (m), 1043 (w), 1013 (m), 976 (m), 951 (w), 910 (m), 883 (w), 837 (s), 818 (s), 799 (m), 775 (m), 756 (s), 731 (s), 718 (m), 704 (m), 692 (m), 673 (m), 656 (m), 604 (m). HR-MS (ESI) calcd. for [C_30_H_29_NO_3_SSi + H]^+^: 512.1637; Found: 512.1719. Anal. calcd. for C_30_H_29_NO_3_SSi [511.71]: C 70.42, H 5.71, N 2.74, S 6.27; Found: C 70.22, H 5.79, N 2.70, S 6.20.

### 3.3. Synthesis of 2-(4-((tetrahydro-2H-pyran-2-yl)oxy)phenyl)-4-(4-((2-(trimethylsilyl)ethyl)-thio)phenyl)pyrano[2,3-b]indole *(**3b**)*

To a degassed solution of **1** (0.24 g, 0.50 mmol) in triethylamine/1,4-dioxane (1:1, 5 mL) was added PdCl_2_(PPh_3_)_2_ (0.018 g, 0.025 mmol), CuI (0.0048 g, 0.025 mmol), and alkyne **2b** (0.111 g, 0.550 mmol) and the mixture was stirred at 20 °C for 4 h. Then, the reaction mixture was stirred at 90 °C for another 18 h. After cooling to room temp the mixture was diluted with water (50 mL) and extracted with ethyl acetate (4 × 50 mL). The combined organic layers were then washed with a saturated solution of aqueous NH_4_Cl (2 × 100 mL) and dried with anhydrous Na_2_SO_4_. After filtration, the solvent was removed under reduced pressure and the crude product was purified by column chromatography on silica gel (cyclohexane/ethyl acetate (1:1)) and recrystallized in *n*-hexane/ethyl acetate (3:1) to give compound **3b** (0.201 g, 0.363 mmol, 73%) as an orange solid, Mp 135 °C. R*_f_* (*n*-hexane/ethyl acetate (1:1)): 0.36.

^1^H NMR (300 MHz, CDCl_3_): *δ* 0.11 (s, 9 H), 1.01–1.07 (m, 2 H), 1.60–1.79 (m, 3 H), 1.88–1.95 (m, 2 H), 1.97–2.08 (m, 1 H), 3.08–3.13 (m, 2 H), 3.62–3.69 (m, 1 H), 3.85–3.94 (m, 1 H), 5.53 (t, *^3^J_H-H_* = 2.8 Hz, 1 H), 7.07–7.14 (m, 2 H), 7.17–7.20 (m, 2 H), 7.44–7.50 (m, 3 H), 7.72–7.78 (m, 4 H), 8.02 (d, *^3^J_H-H_* = 8.7 Hz, 2 H). ^13^C NMR (75 MHz, CDCl_3_): *δ* 1.5 (CH_3_), 16.9 (C_quat_), 18.8 (CH_2_), 25.3 (CH_2_), 29.0 (CH_2_), 30.0 (CH_2_), 62.3 (CH_2_), 80.0 (CH), 97.0 (C_quat_), 103.7 (CH_2_), 117.2 (CH), 119.3 (CH), 121.6 (CH), 122.3 (CH), 122.6 (CH), 123.0 (C_quat_), 125.0 (CH), 127.9 (CH), 128.1 (C_quat_), 129.0 (CH), 129.1 (CH), 133.4 (C_quat_), 141.7 (C_quat_), 156.8 (C_quat_), 159.9 (C_quat_), 170.9 (C_quat_), 173.3 (C_quat_). IR (ATR):*ῦ* [cm^−1^] 3046 (w), 2941 (w), 2922 (w), 2852 (w), 1632 (m), 1607 (w), 1593 (m), 1522 (s), 1504 (s), 1468 (m), 1441 (s), 1422 (s), 1379 (m), 1346 (w), 1327 (w), 1285 (w), 1240 (s), 1196 (m), 1177 (m), 1146 (w), 1128 (w), 1101 (m), 1072 (m), 1063 (m), 1032 (s), 1007 (m), 957 (w), 939 (w), 920 (w), 905 (w), 876 (m), 858 (m), 820 (s), 806 (m), 777 (m), 760 (s), 750 (m), 721 (m), 706 (m), 694 (m), 677 (w), 640 (w), 613 (m). HR-MS (ESI) calcd. for [C_33_H_35_NO_3_SSi + H]^+^: 554.2107; Found: 554.2180. Anal. calcd. for C_33_H_35_NO_3_SSi [553.79]: C 71.57, H 6.37, N 2.53, S 5.79; Found: C 71.65, H 6.39, N 2.58, S 5.84.

### 3.4. Synthesis of 4-(4-(4-((2,5,8,11,15,18,21,24-octaoxapentacosan-13-yl)thio)phenyl)-pyrano[2,3-b]indol-2-yl)phenol *(**5**)*

A mixture of **3a** (0.760 g, 1.49 mmol), KF (0.720 g, 12.4 mmol) and molecular sieves (15 pellets, 3 Å) in dry DMF (19 mL) was stirred at 100 °C for 19 h. After cooling to room temp, **4** (0.667 g, 1.24 mmol) was added and the suspension was stirred at 90 °C for 23 h. After cooling to room temp, water (30 mL) was added, and the mixture was filtrated and rinsed with ethyl acetate (50 mL). The aqueous layer was extracted with ethyl acetate (3 × 150 mL). The combined organic layers were washed with brine (150 mL) and dried with anhydrous Na_2_SO_4_. After filtration, the solvent was removed under reduced pressure and the crude product was purified twice by column chromatography on silica gel (petroleum ether/dichloromethane/ethyl acetate/methanol (5:3:1:0.3 to 5:3:1:0.9)) to give compound **5** (0.270 g, 0.367 mmol, 30%) as a red oil. R*_f_* (dichloromethane/methanol (20:1)): 0.45.

^1^H NMR (300 MHz, CDCl_3_): *δ* 2.08 (s, 1 H), 3.36 (s, 6 H), 3.52–3.55 (m, 4 H), 3.65–3.69 (m, 21 H), 3.79–3.81 (m, 4 H), 7.18 (t, *^3^J_H-H_* = 7.7 Hz, 2 H), 7.34 (d, *^3^J_H-H_* = 8.6 Hz, 2 H), 7.49 (t, *^3^J_H-H_* = 7.7 Hz, 1 H), 7.66 (d, *^3^J_H-H_* = 8.2 Hz, 2 H), 7.76-7.80 (m, 2 H), 7.90 (d, *^3^J_H-H_* = 8.6 Hz, 2 H). ^13^C NMR (75 MHz, CDCl_3_): *δ* 1.2 (CH_2_), 29.8 (CH_3_), 47.6 (CH_2_), 59.2 (CH_2_), 70.6 (CH), 70.7 (CH), 70.8 (CH), 70.8 (CH), 70.9 (CH), 71.0 (CH), 72.1 (CH), 103.8 (CH_2_), 116.2 (C_quat_), 117.4 (CH_2_), 118.3 (CH_2_), 121.9 (C_quat_), 122.0 (CH), 122.2 (C_quat_), 122.3 (CH), 128.7 (CH_2_), 128.9 (CH), 129.2 (C_quat_), 130.2 (C_quat_), 134.0 (C_quat_), 139.9 (C_quat_), 149.1 (C_quat_), 158.3 (C_quat_), 161.8 (C_quat_). IR (ATR):*ῦ* [cm^−1^] 3138 (w), 3076 (w), 2871 (m), 2814 (w), 2683 (w), 2598 (w), 2490 (w), 2423 (w), 1632 (m), 1605 (m), 1589 (m), 1574 (w), 1526 (m), 1506 (m), 1468 (m), 1441 (m), 1429 (m), 1379 (m), 1352 (w), 1327 (w), 1288 (m), 1242 (m), 1211 (w), 1196 (m), 1177 (s), 1099 (s), 1086 (s), 1028 (m), 1015 (m), 978 (m), 957 (m), 934 (w), 864 (w), 820 (m), 775 (m), 756 (m), 741 (w), 719 (w), 704 (m), 675 (w), 660 (w), 642 (w), 629 (w). HR-MS (ESI) calcd. for [C_40_H_49_NO_10_S] + H^+^: 736.3150; Found: 736.3152. Anal. calcd. for C_40_H_49_NO_10_S [735.89]: C 65.29, H 6.71, N 1.90, S 4.36; Found: C 65.01, H 6.31, N 1.87, S 3.98.

### 3.5. Synthesis of 4-(4-((2,5,8,11,15,18,21,24-octaoxapentacosan-13-yl)thio)phenyl)-2-(4-((tetrahydro-2H-pyran-2-yl)oxy)phenyl)pyrano[2,3-b]indole *(**6**)*

A mixture of **3b** (0.614 g, 1.20 mmol), KF (0.720 g, 12.4 mmol) and molecular sieves (15 pellets, 3 Å) in dry DMF (15 mL) was stirred at 100 °C for 19 h. After cooling to room temp, **4** (0.581 g, 10.0 mmol) was added and the suspension was stirred at 90 °C for 23 h. After cooling to room temp again, water (30 mL) was added, and the mixture was filtrated and rinsed with ethyl acetate (50 mL). The aqueous layer was extracted with ethyl acetate (3 × 150 mL). The combined organic layers were washed with brine (150 mL) and dried with anhydrous Na_2_SO_4_. After filtration, the solvent was removed under reduced pressure and the crude product was purified twice by column chromatography on silica gel (petroleum ether/dichloromethane/ethyl acetate/methanol (5:3:1:0.3 to 5:3:1:0.9)) to give compound **6** (0.645 g, 0.786 mmol, 79%) as a red oil. R*_f_* (petroleum ether/ethyl acetate/dichloromethane/methanol (5:3:1:0.3)): 0.48.

^1^H NMR (600 MHz, CDCl_3_): *δ* 1.66–1.74 (m, 3 H), 1.88–1.92 (m, 2 H), 2.00–2.04 (m, 1 H), 3.37 (s, 6 H), 3.52–3.54 (m, 4 H), 3.62–3.68 (m, 21 H), 3.78–3.82 (m, 4 H), 3.84–3.87 (m, 1 H), 3.88–3.90 (m, 1 H), 5.54 (t, *^3^J_H-H_* = 2.8 Hz, 1 H), 7.13 (t, *^3^J_H-H_* = 6.0 Hz, 1 H), 7.19 (d, *^3^J_H-H_* = 9.4 Hz, 1 H), 7.32 (d, *^3^J_H-H_* = 7.9 Hz, 2 H), 7.49 (t, *^3^J_H-H_* = 7.5 Hz, 1 H), 7.64 (d, *^3^J_H-H_* = 8.5 Hz, 2 H), 7.73–7.76 (m, 2 H), 7.81 (d, *^3^J_H-H_* = 8.3 Hz, 2 H), 8.04 (d, *^3^J_H-H_* = 8.9 Hz, 2 H). IR (ATR):*ῦ* [cm^−1^] 3049 (w), 2938 (w), 2868 (w), 1697 (w),1634 (m), 1603 (m), 1541 (m),1526 (m), 1504 (s), 1468 (m), 1439 (m), 1422 (m), 1371 (m), 1356 (m), 1323 (w), 1283 (m), 1240 (s), 1194 (m), 1177 (s), 1098 (s), 1049 (m), 1036 (s), 1022 (m), 955 (s), 916 (s), 872 (m), 820 (s), 777 (m), 758 (m), 735 (m), 704 (m), 640 (m). HR-MS (ESI) calcd. for [C_45_H_57_NO_11_S + H]^+^: 820.3725; Found: 820.3726. Anal. calcd. for C_45_H_57_NO_11_S [819.37]: C 65.91, H 7.01, N 1.71, S 3.91; Found: C 66.06, H 6.53, N 1.79, S 4.22.

### 3.6. Alternative Synthesis of 4-(4-(4-((2,5,8,11,15,18,21,24-octaoxapentacosan-13-yl)thio)phenyl)-pyrano[2,3-b]indol-2-yl)phenol *(**5**)*

A mixture of **6** (0.840 g, 1.02 mmol) and PTSA monohydrate (0.019 g, 1.02 mmol) in methanol (1.5 mL) and water (2.0 mL) was stirred at room temp for 24 h. Afterwards, dichloromethane (50 mL) was added, and the mixture was washed with a saturated solution of NaHCO_3_ (2 × 100 mL). The aqueous layers were combined and extracted with dichloromethane (2 × 100 mL). Subsequently, the organic layers were combined, washed with brine (100 mL), and dried with anhydrous Na_2_SO_4_. After filtration, the solvent was removed under reduced pressure and the crude product was purified twice by column chromatography on silica gel (petroleum ether/dichloromethane/ethyl acetate/methanol (5:3:1:0.3 to 5:3:1:0.9)) to give compound **5** (0.136 g, 0.186 mmol, 18%) as a red oil.

The NMR data were identical with the sample prepared under Section 3.4.

## 4. Conclusions

The Pd/Cu-catalyzed domino insertion-coupling-cycloisomerization of alkynoyl *o*-iodo anilides and terminal arylacetylenes furnishes a novel 2,4-diarylpyrano[2,3-*b*]indole, which is employed as a substrate for the preparation of a versatile amphiprotic turn-ON luminophore using a fluoride mediated double deprotection-alkylation process in a one-pot fashion. Solubility screenings reveal that the chemosensor can be used in most of organic solvents including DMSO, propan-2-ol and ethanol. Furthermore, aqueous mixtures of up to a 75:25 vol-% water-co-solvent ratio are accessible. The photophysical properties of this hydroxy-2,4-diarylpyrano[2,3-*b*]indole are dependent on the pH of the solvent system. Here, yellow or red luminescence is turned on under acid or basic conditions, respectively, allowing a sensitive read-out against a dark background and a reversible fine tuning of the absorption and emission characteristics. As an improvement over the previous second generation of 2,4-diarylpyrano[2,3-*b*]indoles, higher quantum yields of up to *Φ_f_* = 0.058 and *Φ_f_* = 0.059 in propan-2-ol for the protonated and deprotonated species respectively have been detected. In an aqueous mixture of 75:25 vol-% water/co-solvent ratio, clusters of approximately 140 nm in diameter are formed. These clusters start to rapidly form aggregates at higher concentrations of water. Further studies on the formation of clusters in solution as well as their application in biological systems are currently underway.

## Figures and Tables

**Table 1:** Solubility screening of 2,4-diarylpyrano[2,3-*b*]indole **5** in different organic solvents (*c* = 1.3 × 10^−3^ mol·L^−1^). Solvents were sorted with increasing polarity according to Reichardt’s polarity scale [65].

Solvent	Solubility of Compound 5
cyclohexane	–
*n*-pentane	–
*n*-hexane	–
toluene	+
benzene	+
diethyl ether	–
methyl tert-butyl ether	–
1,4-dioxane	+
tetrahydrofuran	+
ethyl acetate	+
chloroform	+
dichloromethane	+
acetone	+
DMF	+
dimethyl sulfoxide	+
acetonitrile	+
propan-2-ol	+
ethanol	+
methanol	+
water	–

**Table 2:** Solubility screening of 2,4-diarylpyrano[2,3-*b*]indole **5** in different aqueous systems (*c* = 1.3 × 10^−3^ mol·L^−1^).

Solvent	Solubility of Compound 5
water	–
buffer, HEPES, 0.1 mol·L^−1^, pH 8	–
buffer, MOPS, 0.1 mol·L^−1^, pH 7	–
water, HCl_(aq)_, pH 1	+
water, HCl_(aq)_, pH 3	–
water, pH 7	–
water, NaOH_(aq)_, pH 11	–
water, NaOH_(aq)_, pH 13	+
tetrahydrofuran/water	25:75
dimethyl sulfoxide/water	70:30
propan-2-ol/water	35:65

**Table 3:** Photophysical data of 2,4-diarylpyrano[2,3-*b*]indole **5** recorded in propan-2-ol (*c*_0_(**5**) = 10^−5^–10^−7^ mol·L^−1^) at *T* = 293 K. Protonated and deprotonated spectra were recorded with an excess amount of TFA or DBU (*c*(TFA/DBU) = 10^−2^ mol·L^−1^).

Compound	*λ_max,abs_* [nm]	*λ_max,em_* [nm]	*Stokes* Shift Δ*ῦ*	Quantum Yield
	(*ε*) [L·cm^−1^·mol^−1^]		[cm^−1^]	*Φ_f_*
**5**	372 (14,200)	-	-	<0.010
**5 + H^+^** ^[a]^	455 (22,400)	539	3400	0.039 ^[b]^
				0.058 ^[c]^
**5 − H^+^** ^[a]^	533 (24,500)	628	2800	0.059 ^[c]^

^[a]^ Protonation by addition of excess TFA or DBU (10 equivs). ^[b]^ Determined with Coumarin 153 in ethanol (*Φ_f_* = 0.38) as standard [69]. ^[c]^Absolute quantum yield.

**Table 4:** Photophysical data of 2,4-diarylpyrano[2,3-*b*]indole **5**, **5 + H^+^**, and **5 − H^+^** recorded in propan-2-ol/water (*c*_0_(**5**) = 10^−5^–10^−7^ mol·L^−1^) at *T* = 293 K. Protonated and deprotonated spectra were recorded with an excess amount of TFA or DBU (*c*(TFA/DBU) = 10^−2^ mol·L^−1^).

Compound	*λ_max,abs_* [nm]	*λ_max,em_*[nm]	*Stokes* Shift Δ*ῦ*	Quantum Yield
	(*ε*) [L·cm^−1^·mol^−1^]		[cm^−1^]	*Φ_f_*
**5**	373 (24200)	-	-	<0.010
**5 + H^+^** ^[a]^	447 (37900)	543	4000	0.014 ^[b]^
**5 − H^+^** ^[a]^	509 (34500)	624	3600	0.018 ^[b]^

^[a]^ Protonation by addition of excess TFA or DBU (10 equivs). ^[b]^ Absolute quantum yield.

## Data Availability

All data of this work are included in the manuscript and the Supplementary Materials. UV/VIS and fluorescence spectra can be provided from the authors upon request.

## Supplementary Materials

The following supporting information can be downloaded at: https://www.mdpi.com/article/10.3390/molecules27072354/s1, Supporting Information: Synthetic and analytic details, and ^1^H and ^13^C NMR spectra of compounds **3**, **5**, and **6**, UV/Vis and fluorescence analyses, and dynamic light scattering analysis.
