# Supplementary material for: Amphipolar, Amphiphilic 2,4-diarylpyrano[2,3-b]indoles as Turn-ON Luminophores in Acidic and Basic Media
Source: Molecules. 2022 Apr 6;27(7):2354. doi: 10.3390/molecules27072354 (PMC9000430; doi:10.3390/molecules27072354)
Supplement: Supplementary file 1 [file molecules-27-02354-s001.zip › molecules-1646095-supplementary.pdf]

Supplementary Materials

Amphipolar, Amphiphilic 2,4-diarylpyrano[2,3-b]indoles as Turn-ON Luminophores in Acidic and Basic Media

Tobias Wilcke [1], Alexandru Postole [1], Marcel Krüsmann [2], Matthias Karg [2] and Thomas J. J. Müller [1,*]

[1] Institut für Organische Chemie und Makromolekulare Chemie,
Heinrich-Heine-Universität Düsseldorf,
Universitätsstraße 1, D-40225 Düsseldorf, Germany;
ThomasJJ.Mueller@uni-duesseldorf.de

[2] Institut für Physikalische Chemie,
Heinrich-Heine-Universität Düsseldorf,
Universitätsstraße 1, D-40225 Düsseldorf, Germany

*To whom the correspondence should be addressed.

T.Wilcke@hhu.de; Alexandru.Postole@su.se; marcel.kruesmann@hhu.de; karg@hhu.de

Table of Contents

1. ^{1}H and ^{13}C NMR Spectra 2

1.1. 4-(4-(4-((2-(Trimethylsilyl)ethyl)thio)phenyl)pyrano[2,3-*b*]indol-2-yl)phenyl acetate (**3a**) 2

1.2. 2-(4-((Tetrahydro-2*H*-pyran-2-yl)oxy)phenyl)-4-(4-((2-(trimethylsilyl)ethyl)thio)-phenyl)pyrano[2,3-*b*]indole (**3b**) 3

1.3. 4-(4-(4-((2,5,8,11,15,18,21,24-Octaoxapentacosan-13-yl)thio)phenyl)-pyrano[2,3-*b*]indol-2-yl)phenol (**5**) 4

1.4. 4-(4-((2,5,8,11,15,18,21,24-octaoxapentacosan-13-yl)thio)phenyl)-2-(4-((tetrahydro-2*H*-pyran-2-yl)oxy)phenyl)pyrano[2,3-*b*]indole (**6**) 5

2. Determination of pK_a values 6

3. UV/Vis measurements in THF 9

4. UV/Vis measurements in THF/water (25:75) 10

5. UV/Vis measurements in propan-2-ol/water (5:95) 12

6. Dynamic light scattering analysis 12

7. References 14

1. ^{1}H and ^{13}C NMR Spectra

1.1. 4-(4-(4-((2-(Trimethylsilyl)ethyl)thio)phenyl)pyrano[2,3-*b*]indol-2-yl)phenyl acetate (3a)

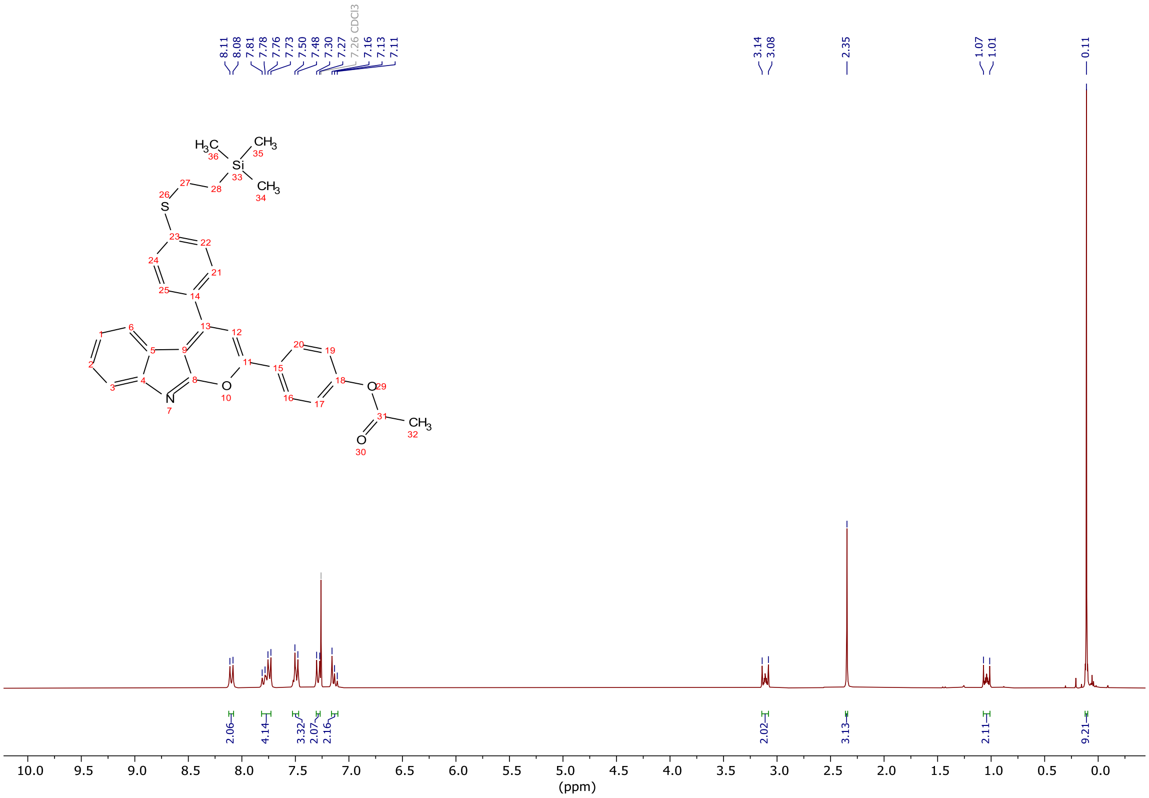

Figure S1. ^{1}H NMR spectrum (300 MHz) of **3a**; recorded in $CDCl_3$ at 293 K.

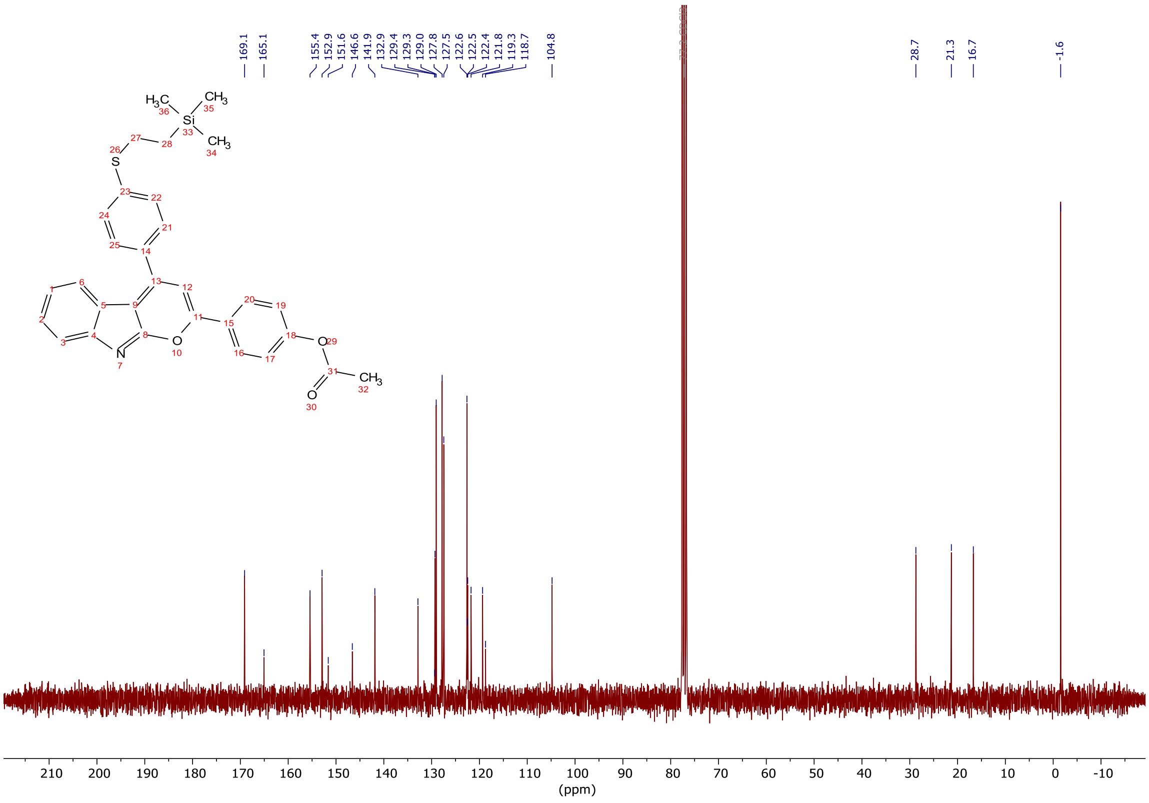

Figure S2. ^{13}C NMR spectrum (75 MHz) of **3a**; recorded in $CDCl_3$ at 293 K.

1.2. 2-(4-((Tetrahydro-2*H*-pyran-2-yl)oxy)phenyl)-4-(4-((2-(trimethylsilyl)ethyl)thio)-phenyl)pyrano[2,3-*b*]indole (3b)

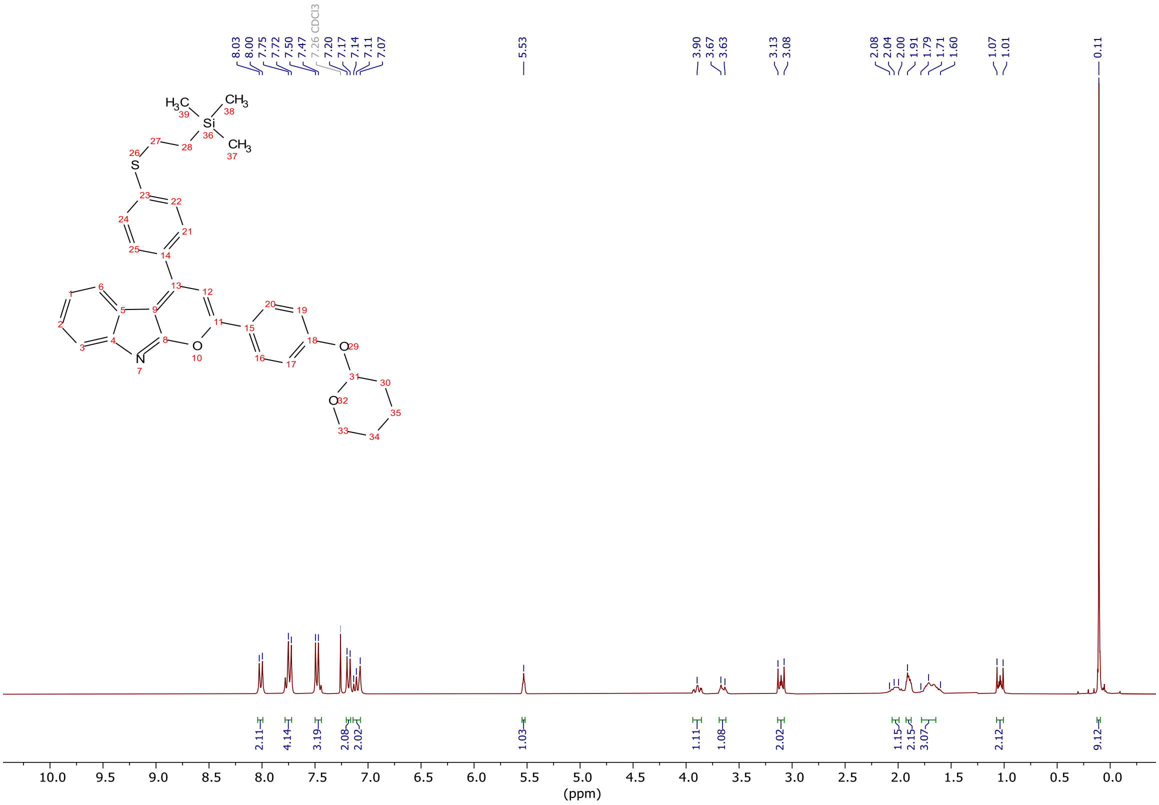

Figure S3. ^{1}H NMR spectrum (300 MHz) of **3b**; recorded in $CDCl_3$ at 293 K.

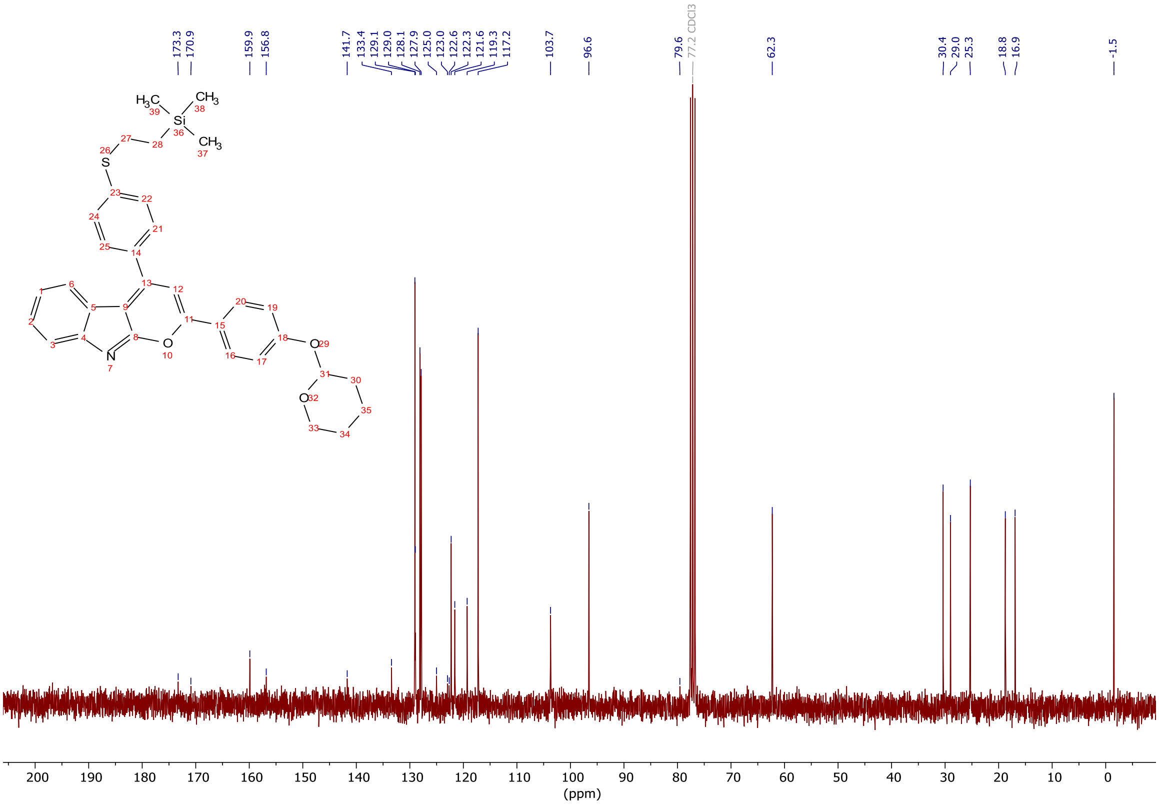

Figure S4. ^{13}C NMR spectrum (75 MHz) of **3b**; recorded in $CDCl_3$ at 293 K.

1.3. 4-(4-(4-((2,5,8,11,15,18,21,24-Octaoxapentacosan-13-yl)thio)phenyl)-pyrano[2,3-*b*]indol-2-yl)phenol (5)

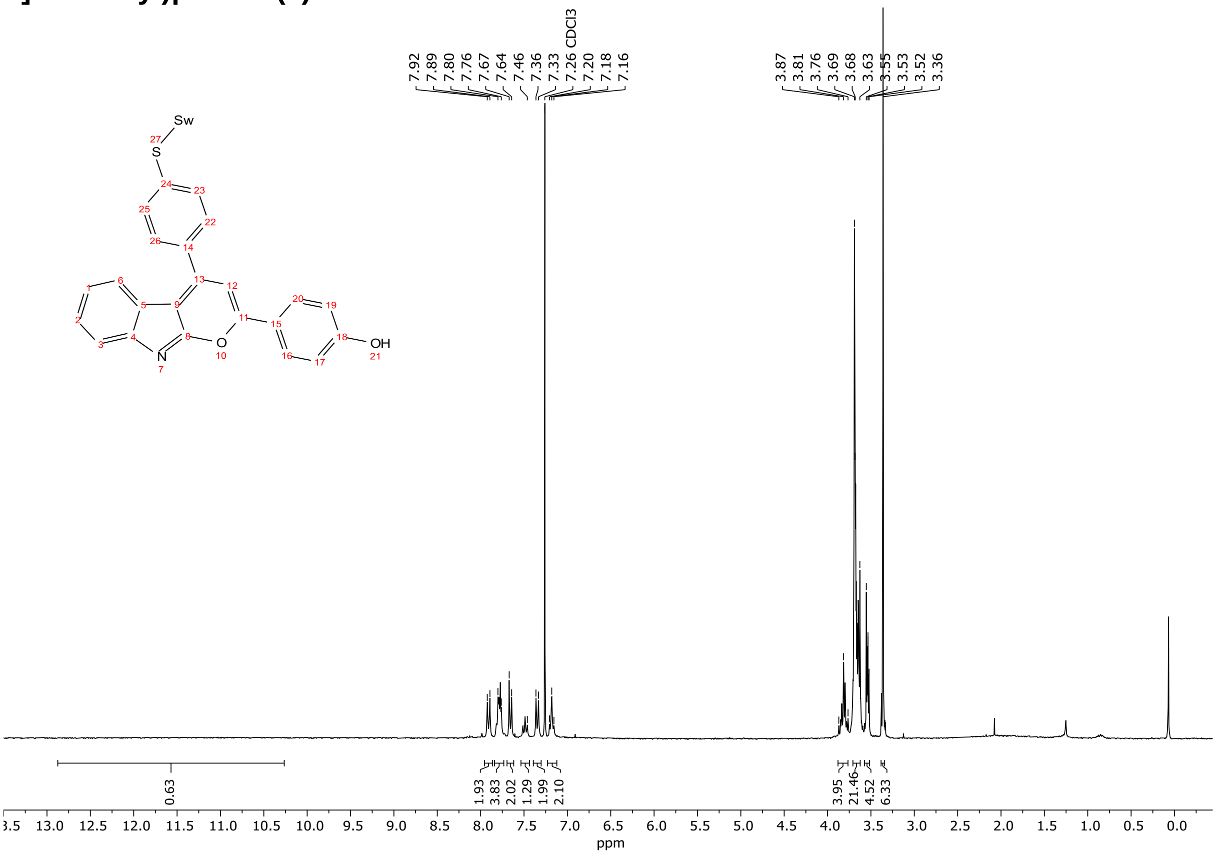

Figure S5. ^{1}H NMR spectrum (300 MHz) of **5**; recorded in $CDCl_3$ at 293 K.

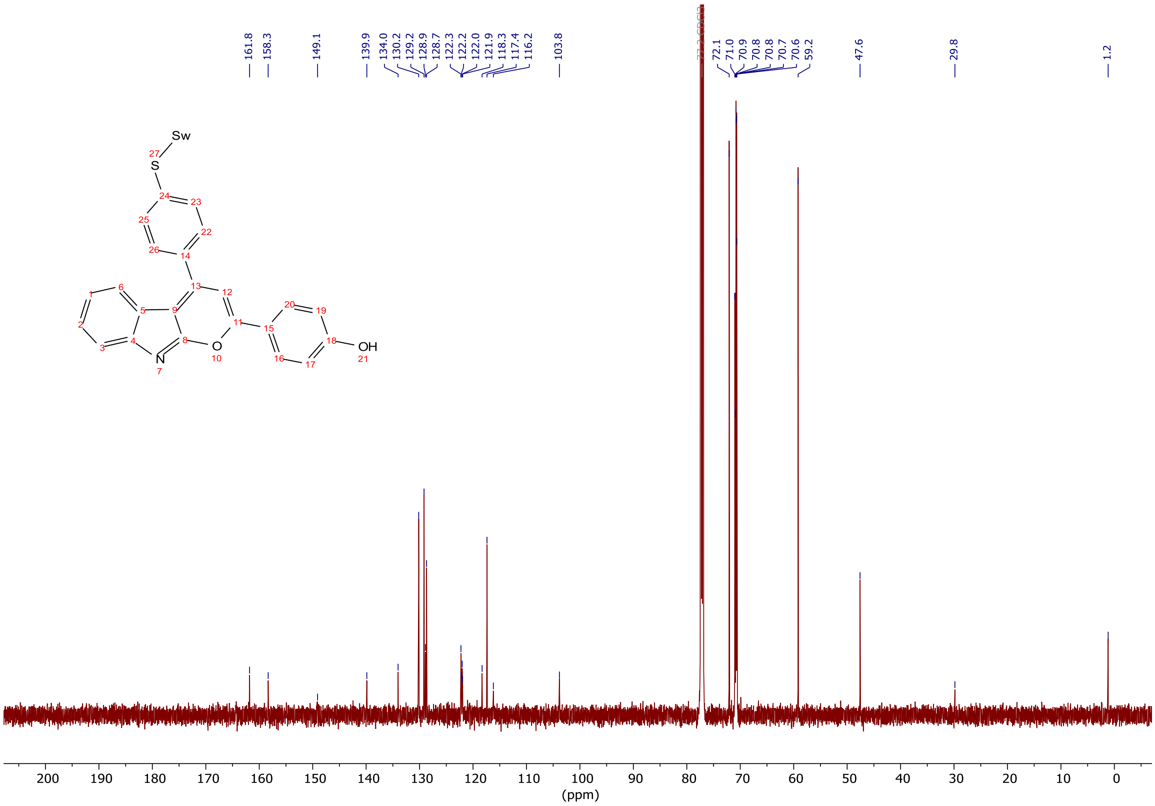

Figure S6. ^{13}C NMR spectrum (75 MHz) of **5**; recorded in $CDCl_3$ at 293 K.

1.4. 4-(4-((2,5,8,11,15,18,21,24-octaoxapentacosan-13-yl)thio)phenyl)-2-(4-((tetrahydro-2*H*-pyran-2-yl)oxy)phenyl)pyrano[2,3-*b*]indole (6)

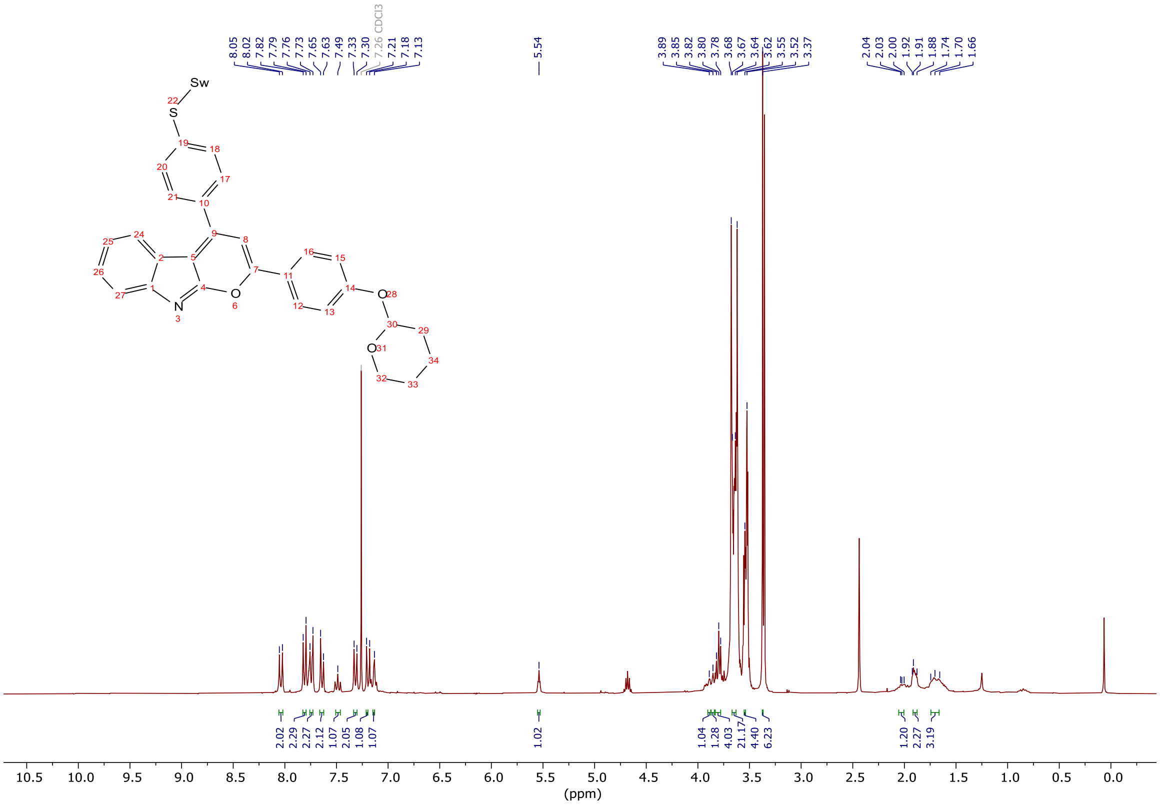

Figure S7. ^{1}H NMR spectrum (300 MHz) of **6**; recorded in $CDCl_3$ at 293 K.

2. Determination of pK_a values

For the determination of pK_{a1} of 2,4-diarylpyrano[2,3-*b*]indole **5**, absorption spectra at different pH values from 7.0 to 1.3 through addition of TFA were recorded in propan-2-ol (Figure S8).

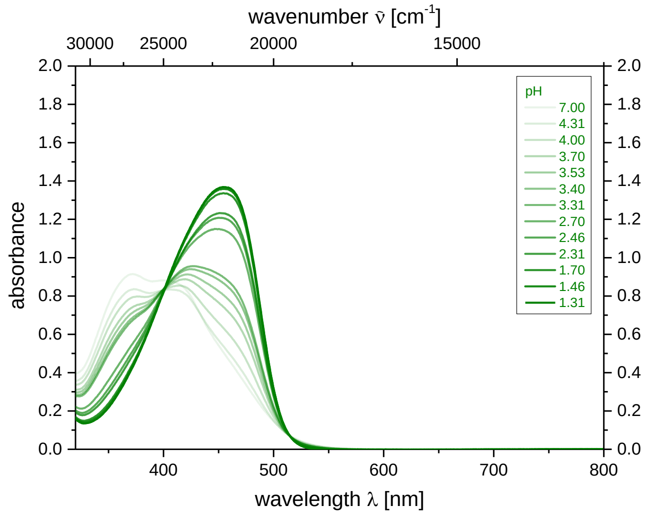

Figure S8. Absorption spectra of **5** (c_0(**5**) = 5.0 · 10^{-5} mol·L^{-1}) at different concentrations of trifluoroacetic acid (recorded in propan-2-ol at T = 293 K).

Afterwards, absorbance difference of these spectra referenced to a neutral spectrum at pH 7 was plotted against wavelength to determine the two wavelengths where a change in absorbance was most pronounced ($\lambda_{abs,min}$ = 357 nm, $\lambda_{abs,max}$ = 465 nm, Figure S9).

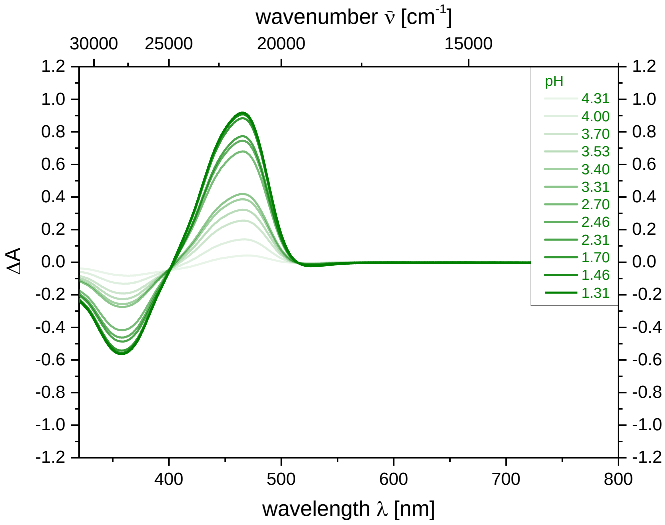

Figure S9. Absorbance difference spectra of **5** (c_0(**5**) = 5.0 · 10^{-5} mol·L^{-1}) at various concentrations of trifluoroacetic acid (recorded in propan-2-ol at T = 293 K).

In the last step the change in absorbance at these two specific wavelengths was plotted against the corresponding pH value (Figure S10). Here, the inflection points of either graph correlate with the pK_{a1} value of the indole nitrogen atom, which lie in both cases at around pH 3.5.

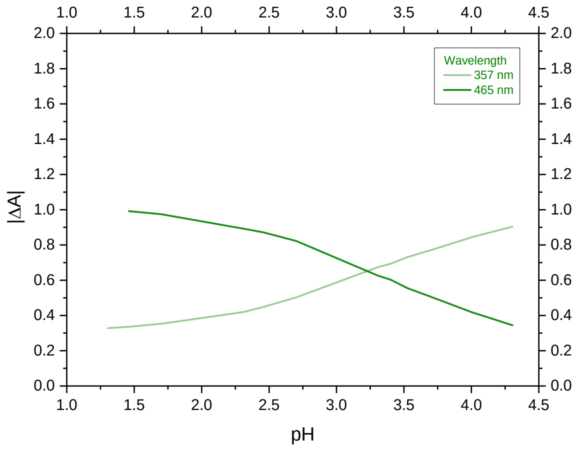

Figure S10. Absorbance variation of **5** (c_0(**5**) = 5.0 · 10^{-5} mol·L^{-1}) versus pH at specific wavelengths ($\lambda_{max,abs}$ = 357 nm and 465 nm) with addition of trifluoroacetic acid (recorded in propan-2-ol at T = 293 K).

In the same way, the pK_{a2} value of the hydroxy group has been determined by deprotonation with DBU as a base. After plotting and evaluating the different types of spectra, inflection points and therefore a pK_{a2} value of 10.5 can be determined for the phenolic hydroxy group of 2,4-diarylpyrano[2,3-*b*]indole **5** (Figures S11-S13).

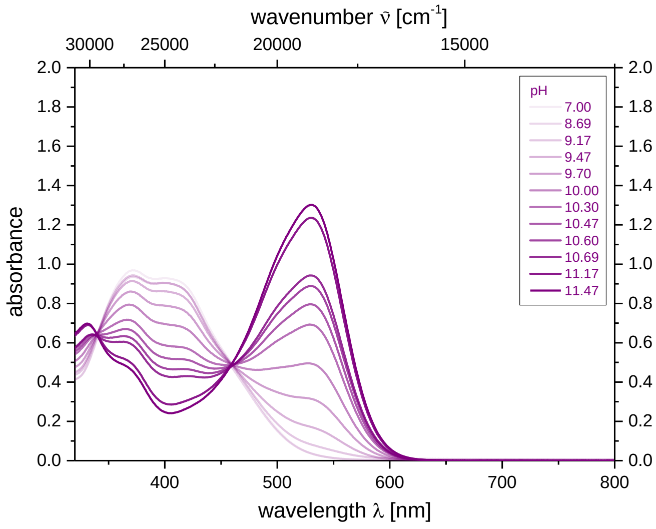

Figure S11. Absorption spectra of **5** (c_0(**5**) = 5.0 · 10^{-5} mol·L^{-1}) at different concentrations of 1,8-diazabicyclo[5.4.0]undec-7-ene (DBU) (recorded in propan-2-ol at T = 293 K).

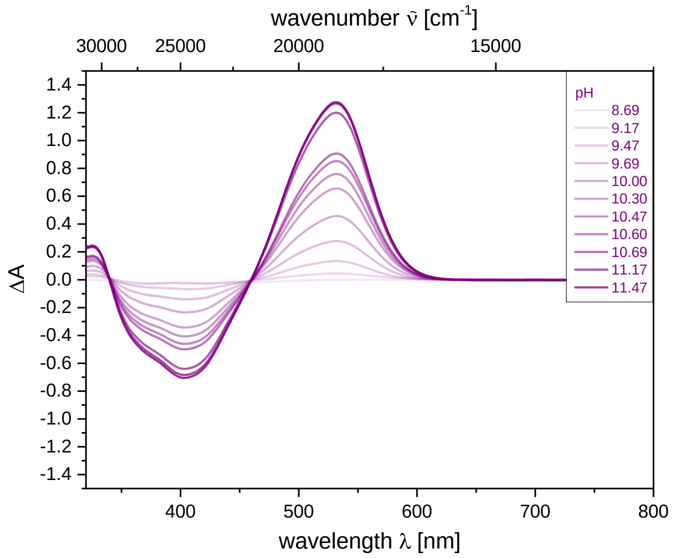

Figure S12. Absorbance difference spectra of **5** (c_0(**5**) = 5.0 · 10^{-5} mol·L^{-1}) at various concentrations of 1,8-diazabicyclo[5.4.0]undec-7-ene (DBU) (recorded in propan-2-ol at T = 293 K).

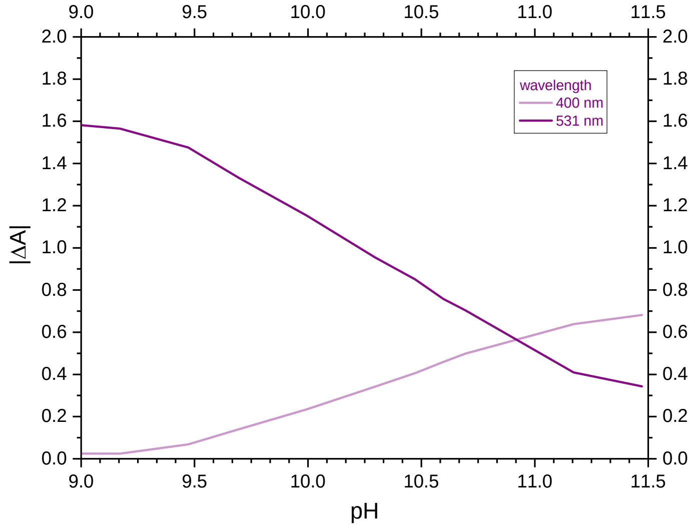

Figure S13. Absorbance variation of **5** (c_0(**5**) = 5.0 · 10^{-5} mol·L^{-1}) versus pH at specific wavelengths ($\lambda_{max,abs}$ = 400 nm, and 531 nm) with 1,8-diazabicyclo[5.4.0]undec-7-ene (DBU) as base (recorded in propan-2-ol at T = 293 K).

3. UV/Vis measurements in THF

Additionally, photophysical properties of 2,4-diarylpyrano[2,3-*b*]indole **5** were investigated in THF as a water miscible organic solvent (Table S1, Figures S14 and S15). The absorption bands of 2,4-diarylpyrano[2,3-*b*]indole **5**, **5 + H**$^+$, and **5 – H**$^+$ are slightly hypsochromically shifted and broader than in propan-2-ol, while emission maxima of **5 + H**$^+$, and **5 – H**$^+$ remained unchanged at around 540 nm and 630 nm, respectively. Given this observation, *Stokes* shifts were marginally higher at $\Delta\tilde{\nu}$ = 4.100 cm^{-1} for both species. The molar decadic attenuation coefficients ε of 2,4-diarylpyrano[2,3-*b*]indole **5** and **5 + H**$^+$ in THF were approximately the same as compared to propan-2-ol, whereas values for **5 – H**$^+$ were less than in propan-2-ol. The absolute quantum yields of the two non-neutral 2,4-diarylpyrano[2,3-*b*]indole species **5 + H**$^+$, and **5 – H**$^+$ in THF were smaller than in propan-2-ol, therefore the latter was chosen as co-solvent for investigations in aqueous media.

Table S1. Photophysical data of 2,4-diarylpyrano[2,3-*b*]indole **5** recorded in THF (c_0(**5**) = 10^{-5} – 10^{-7} mol·L^{-1}) at T = 293 K. Protonated and deprotonated spectra were recorded with an excess amount of TFA or DBU (c(TFA/DBU) = 10^{-2} mol·L^{-1}).

Compound	$\lambda_{max,abs}$ [nm]	(ε) [L·cm^{-1}·mol^{-1}]	$\lambda_{max,em}$ [nm]	*Stokes* shift $\Delta\tilde{\nu}$ [cm^{-1}]	Quantum yield Φ_f
**5**	367	15.600	-	-	<0.001[b]
**5 + H**$^{+[a]}$	443	26.900	541	4.100	0.026[b]
**5 – H**$^{+[a]}$	500	12.400	629	4.100	0.005[b]

[a]Protonation or deprotonation by addition of excess TFA or DBU (10.000 eq). [b]Absolute quantum yield.

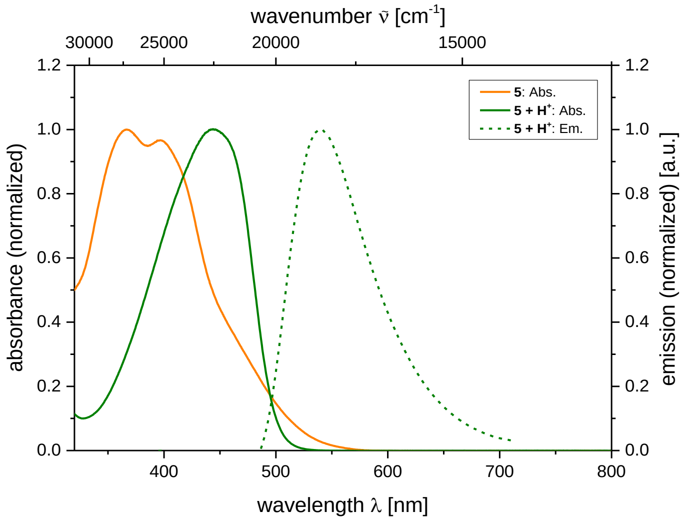

Figure S14. Normalized absorption spectrum of **5** (orange solid line), **5 + H**$^+$ (green solid line), and normalized emission spectrum of **5 + H**$^+$ (green dashed line) (c_0(**5**) = 4.9 · 10^{-5} mol·L^{-1}, c(TFA) = 10^{-2} mol·L^{-1}, recorded in THF at T = 293 K, $\lambda_{exc,\mathbf{5+H^+}}$ = 443 nm).

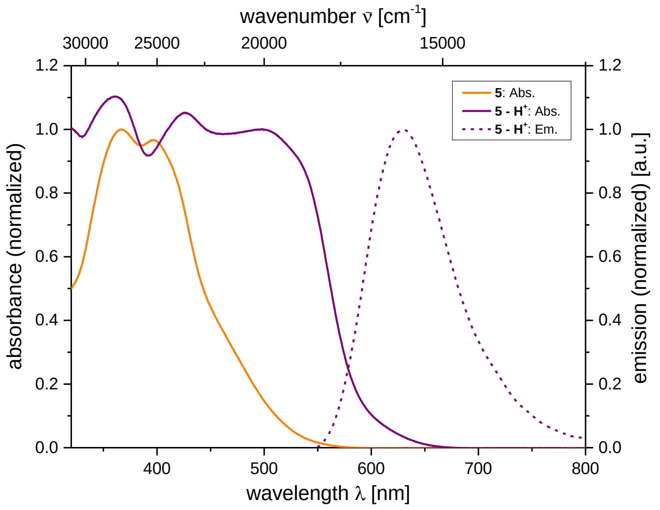

Figure S15. Normalized absorption spectrum **5** (orange solid line), **5 – H⁺** (purple solid line), and normalized emission spectrum of **5 – H⁺** (purple dashed line) (c_0(**5**) = 4.9 · 10^{-5} mol·L^{-1}, c(DBU) = 10^{-2} mol·L^{-1}, recorded in THF at T = 293 K, $\lambda_{exc,\mathbf{5\text{ - }H+}}$ = 500 nm).

4. UV/Vis measurements in THF/water (25:75)

Furthermore, photophysical properties of 2,4-diarylpyrano[2,3-*b*]indole **5** were investigated in an aqueous mixture of THF/H_2O (25:75) (Figures S16 and S17, Table S2). By exchanging ¾ of the solvent with water, absorption bands of species **5** and **5 – H⁺** are slightly shifted bathochromically. Molar decadic attenuation coefficients ε of **5** and **5 – H⁺** are smaller than in pure THF. *Stokes* shifts $\Delta\tilde{\nu}$ remain mostly unchanged by switching to a more polar solvent mixture at around 4.100 cm^{-1}. The absolute quantum yields of the two non-neutral 2,4-diarylpyrano[2,3-*b*]indole species **5 + H⁺** and **5 – H⁺** in THF/H_2O could not be determined due to solubility issues after addition of TFA and DBU leading to scattering of light.

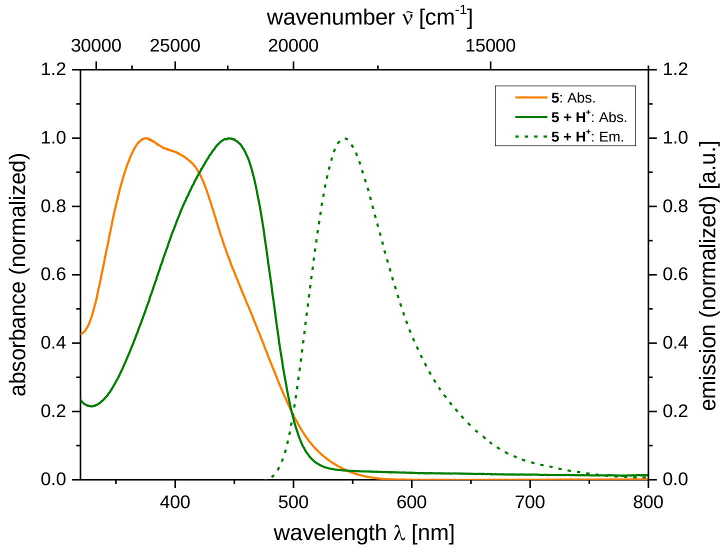

Figure S16. Normalized absorption spectrum of **5** (orange solid line), **5 + H⁺** (green solid line), and normalized emission spectrum of **5 + H⁺** (green dashed line) (c_0(**5**) = 4.6 · 10^{-5} mol·L^{-1}, *c*(TFA) = 10^{-2} mol·L^{-1}, recorded in THF/H_2O (25:75) at T = 293 K, $\lambda_{exc,\mathbf{5+H+}}$ = 445 nm).

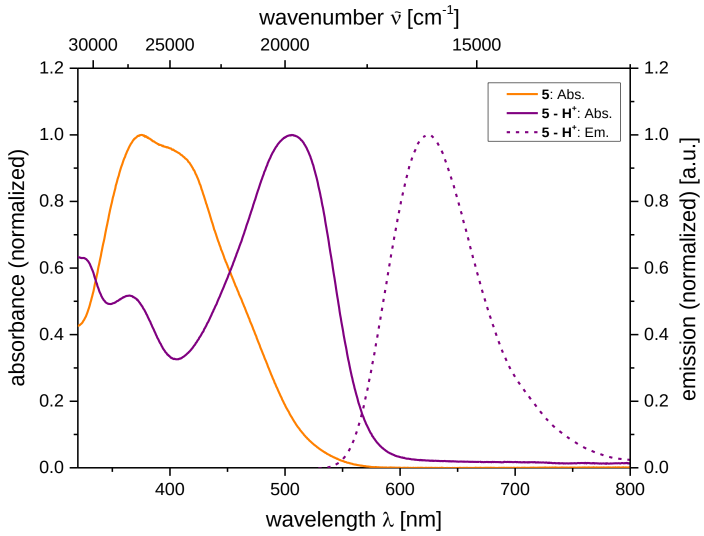

Figure S17. Normalized absorption spectrum **5** (orange solid line), **5 – H⁺** (purple solid line), and normalized emission spectrum of **5 – H⁺** (purple dashed line) (c_0(**5**) = 4.6 · 10^{-5} mol·L^{-1}, *c*(TFA) = 10^{-2} mol·L^{-1}, recorded in THF/H_2O (25:75) at T = 293 K, $\lambda_{exc,\mathbf{5-H+}}$ = 506 nm).

Table S2. Photophysical data of 2,4-diarylpyrano[2,3-*b*]indole **5** recorded in THF/H_2O (25:75) (c_0(**5**) = 10^{-5} – 10^{-7} mol·L^{-1}) at T = 293 K. Protonated and deprotonated spectra were recorded with an excess amount of TFA or DBU (c(TFA/DBU) = 10^{-2} mol·L^{-1}).

Compound	$\lambda_{max,abs}$ [nm]	(ε) [L·cm^{-1}·mol^{-1}]	$\lambda_{max,em}$ [nm]	*Stokes* shift $\Delta\tilde{\nu}$ [cm^{-1}]	Quantum yield Φ_f
**5**	376	12.700	-	-	<0.01 [b]
**5 + H^{+}**[a]	445	12.200	543	4.100	Not measurable
**5 – H^{+}**[a]	506	11.100	625	3.800	Not measurable

[a]Protonation by addition of excess TFA or DBU (1.000 eq). [b]Absolute quantum yield.

5. UV/Vis measurements in propan-2-ol/water (5:95)

Additionally, measurements of a turbid solution with higher water concentration of 95% under neutral conditions were performed. Because of the present clusters, scattering of light is clearly visible at longer wavelengths as well as less pronounced absorption maxima (Figure S18).

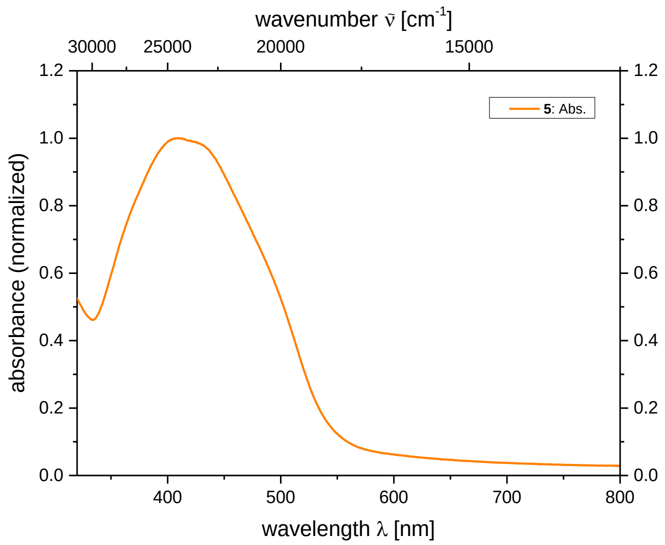

Figure S18. Normalized absorption spectrum (orange solid line) of compound **5** (c_0(**5**) = 6.7 · 10^{-5} mol·L^{-1}, recorded in propan-2-ol/H_2O (5:95) at T = 293 K).

6. Dynamic light scattering analysis

Angle-dependent DLS measurements were performed to study the diffusive behavior of samples with different water contents. The relaxation rate, Γ, was determined using the fast mode data obtained by CONTIN analysis.[1]. Figure S19 shows a plot of the relaxation rate, Γ, as a function of the squared magnitude of the scattering vector, q. Despite some deviations

at larger q^2 we observe linear scaling for all samples. This underlines that we probe translational diffusion. The slopes from the linear fits to the data (solid lines) were used to derive the translational diffusion coefficients, D_T. Hydrodynamic diameter were calculated using the *Stokes-Einstein* equation and are reported in Table S3.

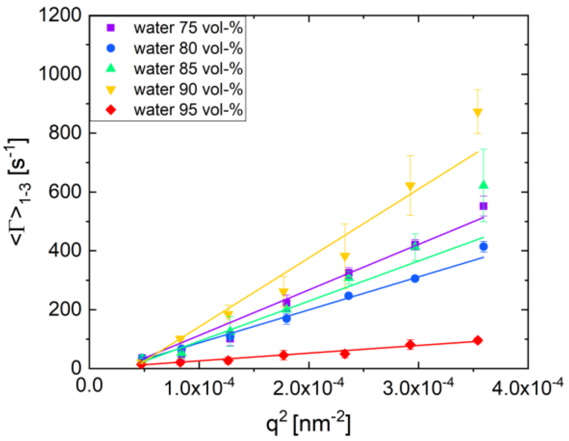

Figure S19. Results from angle-dependent DLS measurements. Plotted is the mean relaxation rate, Γ, as a function of the squared magnitude of the scattering vector q. The solid lines are linear fits to the data.

Figure S20 shows the respective results for the samples that were aged for one month. The hydrodynamic diameter obtained from the data are listed in Table S3. Apart from the sample with the highest water content, all aged samples contain larger clusters as compared to the freshly prepared samples.

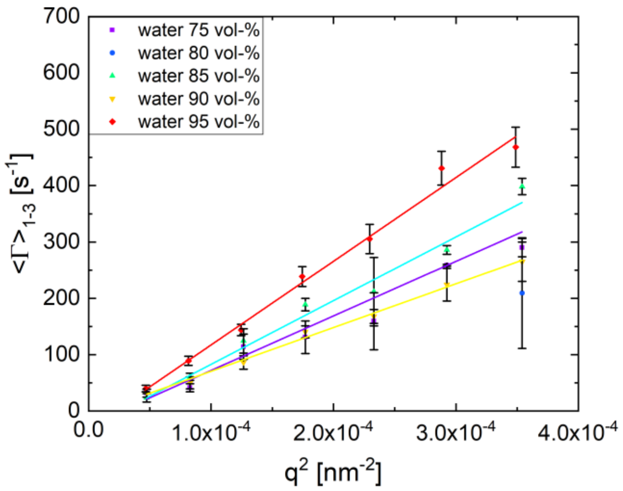

Figure S20. Results from angle-dependent DLS measurements of samples that were aged for one month. Plotted is the mean relaxation rate, Γ, as a function of the squared magnitude of the scattering vector q. The solid lines are linear fits to the data.

Table S3. Hydrodynamic diameters of the clusters in different propan-2-ol water mixtures. Before and after aging for one month.

Water content [vol-%]	Fresh sample hydrodynamic diameter [nm]	Aged sample hydrodynamic diameter [nm]
75	142	260
80	200	–
85	174	268
90	138	466
95	1490	308

7. References

1. Provencher, S. W. *A constrained regularization method for inverting data represented by linear algebraic or integral* equations, Comput. Phys. Commun. **1982,** *27*, 213–227. DOI 10.1016/0010-4655(82)90173-4
